# WARA-PS: a research arena for public safety demonstrations and autonomous collaborative rescue robotics experimentation

**DOI:** 10.1007/s43684-021-00009-9

**Published:** 2021-11-16

**Authors:** Olov Andersson, Patrick Doherty, Mårten Lager, Jens-Olof Lindh, Linnea Persson, Elin A. Topp, Jesper Tordenlid, Bo Wahlberg

**Affiliations:** 1 Department of Computer and Information Science, Linköping University, Linköping, Sweden; 2 Saab Kockums, Malmö, Sweden; 3 Division of Decision and Control Systems, School of Electrical Engineering and Computer Science, KTH Royal Institute of Technology, Stockholm, Sweden; 4 Department of Computer Science, Lund University, Lund, Sweden; 5 Saab Combitech AB, Linköping, Sweden; 6 Division of Automatic Control, Department of Electrical Engineering, Linköping University, Linköping, Sweden

**Keywords:** Autonomous systems, Intelligent system architectures, Research demonstration arena, Autonomous drones, Autonomous marine vessels, Public safety and security, Collaborative robotics

## Abstract

A research arena (WARA-PS) for sensing, data fusion, user interaction, planning and control of collaborative autonomous aerial and surface vehicles in public safety applications is presented. The objective is to demonstrate scientific discoveries and to generate new directions for future research on autonomous systems for societal challenges. The enabler is a computational infrastructure with a core system architecture for industrial and academic collaboration. This includes a control and command system together with a framework for planning and executing tasks for unmanned surface vehicles and aerial vehicles. The motivating application for the demonstration is marine search and rescue operations. A state-of-art delegation framework for the mission planning together with three specific applications is also presented. The first one concerns model predictive control for cooperative rendezvous of autonomous unmanned aerial and surface vehicles. The second project is about learning to make safe real-time decisions under uncertainty for autonomous vehicles, and the third one is on robust terrain-aided navigation through sensor fusion and virtual reality tele-operation to support a GPS-free positioning system in marine environments. The research results have been experimentally evaluated and demonstrated to industry and public sector audiences at a marine test facility. It would be most difficult to do experiments on this large scale without the WARA-PS research arena. Furthermore, these demonstrator activities have resulted in effective research dissemination with high public visibility, business impact and new research collaborations between academia and industry.

## Introduction

The Wallenberg AI, Autonomous Systems and Software Program (WASP) [1] is a recent large-scale Swedish national endeavor with a research focus on the strategic areas of Artificial Intelligence, Autonomous Systems and Future Software Systems. One of the novel features of the program is the establishment of *research arenas* in diverse areas of interest to the program.

The main objectives of the WASP Research Arenas (WARAs) are to increase the value and relevance of research and to shorten knowledge transfer between academia and industry. The arenas offer engineering support combined with a collaborative environment that strengthens and facilitates collaboration between WASP researchers and industry partners. Through the research arenas, system-level platforms and test-bed scenarios far beyond the reach of individual university labs, are made available for research. The networks around the arenas are also incorporated in other parts of WASP, for example in a WASP graduate school project course, [1].

The focus of this paper is on one of these arenas, the WASP Public Safety Arena (WARA-PS) [2]. Public safety and security [3] is used as a broad umbrella term for activities that target the obviation of danger to the general public and to any public place or public property. Public safety and security is generally a function of government, and includes public safety organizations such as law enforcement, fire and emergency rescue and medical services. The focus of these organizations is to prevent and protect from events that could endanger the safety of the general public. This includes natural or man-made disasters, criminal activity, terrorism, larger scale severe accidents and medical health emergencies such as pandemics.

Recently, there have been radical changes in the way many of these public safety organizations operate due to the advent of new technology such as robotics systems and modern means of information and knowledge transfer via IT solutions. A main driver for such technologies are companies and academia. Public safety and security and the drivers that can enhance and improve solutions in these areas are the main target of WARA-PS.

Some of the major focuses of activity in WARA-PS are emergency rescue using ground, aerial, surface and underwater robotic systems; sophisticated command and control systems; human/robot collaboration and interaction; and monitoring and safety of Swedish waterways. The arena allows for a research and development context in which surface vehicles, drones, underwater vehicles and people can collaborate in a unique way to enhance public safety. Larger scenarios are used as research drivers and include search and rescue missions where autonomous vehicles can get close to drifting boats or navigate in challenging waters, while drones provide situation awareness and search capabilities from above. The arena has close cooperation with government authorities and end-users such as land and sea rescue services.

Many of the participating companies in WARA-PS are involved in the development of products to support public safety and security. Many of the participating universities in WARA-PS have targeted emergency rescue and public safety as application scenarios to test basic research results in AI, autonomous systems and future software systems.

The main contributions of this paper are:

- An overview of the WARA-PS data-to-decisions infrastructure and the corresponding core system architecture. The corresponding state-of-art delegation framework used for planning and execution is described in detail.
- Successful results with demonstrations from two selected research projects on autonomous aerial vehicles are presented. The first one concerns cooperative landing of an unmanned aerial vehicle on an unmanned surface vehicle. The second project deals with planning safe trajectories using machine-learned proximity constraints and informed aerial search for victims.
- Successful experimental results from three selected research activities on autonomous sea vessels are presented. The first one is about sensor fusion for GPS-independent positioning, and the second one concerns human assisted operations using teleoperations. The third project deals with semi-automated image annotation in marine environments.

Researchers and PhD students in the WASP program who are affiliated with WARA-PS have performed research in a broad range of areas ranging from mission critical cloud technology to video compression and video quality assessment. See [4] for a list of publications and PhD theses.

### WARA-PS core team

The WARA-PS Core Team consists of researchers and engineers from both industry and academia. Industrial participants in the core team include:

- Saab Naval (Kockums) [5] – Saab Naval, which is the maritime part of Saab, designs, builds and maintains naval surface vessels and submarines. Two of their experimental systems, used for development of autonomous surface vehicles are the Combat Boat 90 and the Piraya. Both platforms have been used for experimentation in WARA-PS and are considered in more detail later in the paper.
- Saab Air (Aeronautics) [6] – Saab Air, which is the aeronautical part of Saab, is a supplier of aircraft systems, advanced aero-structures and a wide range of support solutions within civil and military aviation.
- Combitech [7] – Combitech is an independent technology consulting company that is also a part of Saab AB [8]. They have a wide range of activities in the area of autonomy such as autonomous mining, command and control systems and simulation systems. A number of their products and knowledge are being integrated in the WARA-PS infrastructure.
- Ericsson Research Data Center (ERDC) – ERDC is a research facility used by thousands of developers in different internal and external collaboration projects. A free cloud environment for WASP researchers is provided. On top of the cloud environment AI frameworks and CI/CD pipelines are offered. Most of the cloud based functionality in WARA-PS is hosted in ERDC as well as simulation models of vehicles and sensors. The data center is also used to store collected datasets which are provided through a Resource Portal.
- Axis Communications [9] – Axis Communications is a company that provides network video and audio solutions for security application. In WARA-PS the Axis research department in Lund, Sweden provides image-based sensors used in various research projects. Surveillance cameras of different types provided by Axis are mounted on some of the WARA-PS USVs.
- UMS Skeldar [10] – UMS Skeldar is a joint venture between Saab and the UMS AERO Group in the area of VTOL unmanned aerial systems. In WARA-PS UMS SKELDAR is part of the Core Team. The company provides domain experience and real use cases for UAVs in WARA-PS scenarios.

The main university participant on the core team supporting infrastructure and architecture development, in addition to UAV activities, is the Artificial Intelligence and Integrated Computer Systems Division, Computer and Information Science Department, Linköping University [11] (IDA-AIICS). Additionally, the following organizations have close collaboration with the WARA-PS core team:

- Swedish Maritime Robotics Centre (SMaRC) [12] – The Swedish Maritime Robotics Centre is a national cross-disciplinary industrial research centre for maritime robotics located at KTH Royal Institute of Technology, Sweden. The main task is to perform research on, and demonstrate, solutions that can contribute to the transition to autonomous intelligent underwater systems. They have a wide range of research activities related to WARA-PS such as autonomous underwater perception, underwater navigation and docking and multi-agent mission planning and docking. One of their demonstrators, the SMaRC long-range and long-endurance maritime USV has been used actively in WARA-PS and is integrated in the larger WARA-PS architecture for future experimentation with collaborative robotics in sea rescue scenarios.
- Swedish Sea Rescue Society (SSRS) [13] – SSRS is a voluntary sea rescue organization with over 76 sea rescue stations and 260 rescue units spread out along the Swedish coast. It has over 2300 volunteers with a goal of answering rescue calls at sea within 15 min of any incident. More recently, SSRS has been teaming with other organizations and companies, such as Airpelago, to modernize their rescue services by using state-of-art autonomous technology such as USVs and UAVs. SSRS participates in WARA-PS as one of several units in our larger rescue scenarios at sea. They also interact with PhD students and researchers to enhance rescue technology at sea.

### WARA-PS infrastructure

WARA-PS supports a diverse environment of technology provided by both industrial and academic partners. These systems are used for data collection, test development, demonstrations and development of research among PhD students, senior researchers at the many participating universities and industrial participants. The systems and resources are currently part of WARA-PS and much effort has been put into the integration of these systems into a larger system of collaborative systems. Each of these resources are considered in more detail in other sections of the paper. A more detailed overview of the WARA-PS infrastructure and core system architecture supporting the infrastructure is provided in Section 2.

### WARA-PS operational environment

**Fig. 1:**
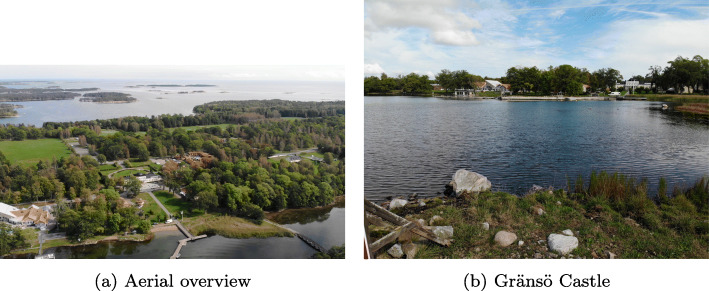
The Gränsö Operational Environment

Gränsö lies about 4 kilometers outside the city of Västervik, Sweden. The operational environment currently used is centered around Gränsö Castle shown in Fig. 1. The harbor and seaside are suitable for researchers to access and test ground, aerial, surface and underwater vehicles.

Västervik also has an airport where the Swedish Drone Center, funded by Vinnova, is active. This center provides support for WARA-PS in the form of dealing with licensing and legal issues regarding use of airspace for flight testing. It also has a large community with access to additional aerial platforms, when needed.

WARA-PS hosts annual workshops in May and September each year where researchers, engineers and project leaders meet to demonstrate progress, collect data and inspire with new challenges and future research issues. During the period between the larger workshops the WARA-PS core team meet here regularly for integration workshops and development of infrastructure. The annual workshop is also open to visitors and media to enhance knowledge transfer to the public.

**Fig. 2:**
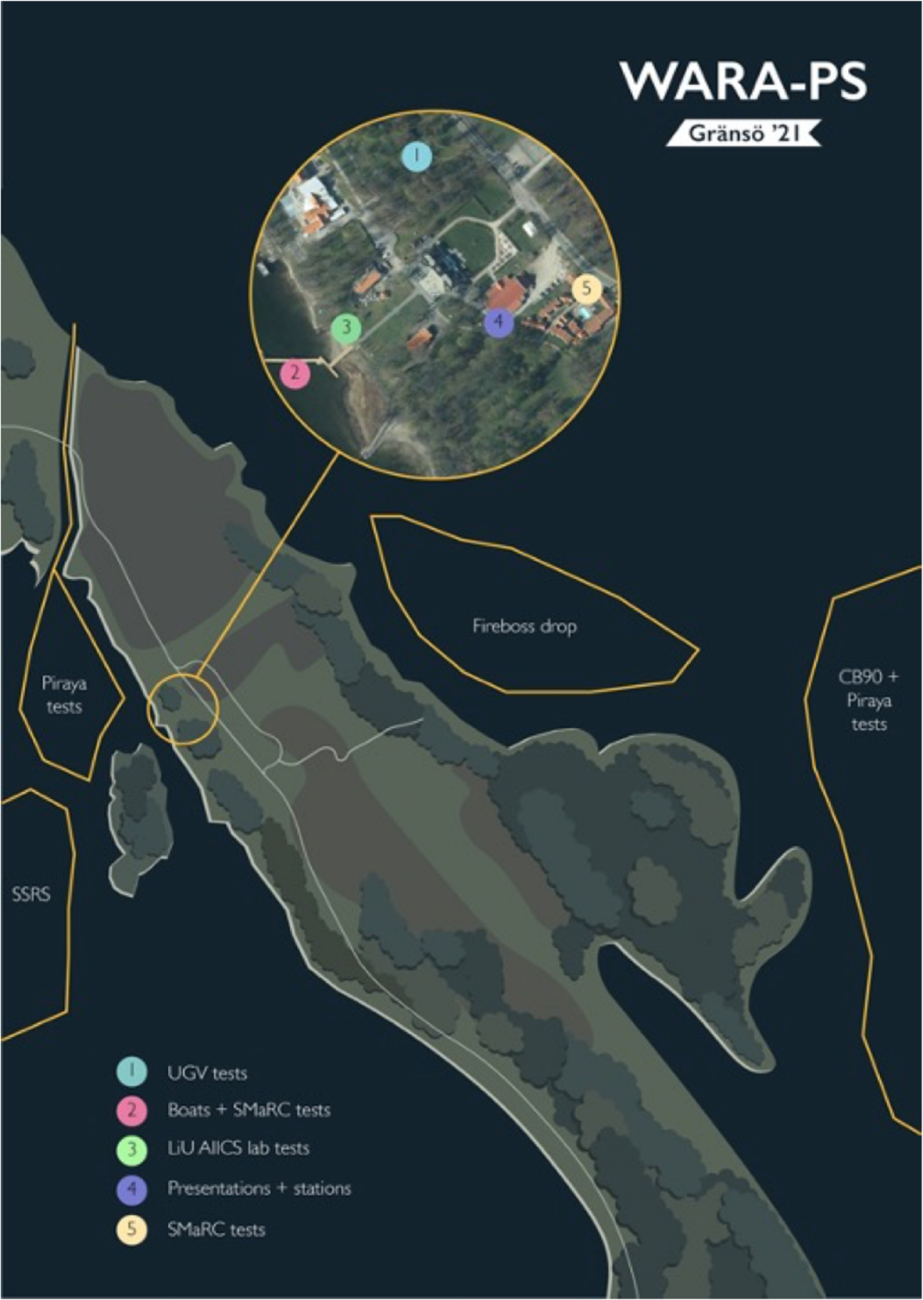
Figurative overview of WARA-PS operational environment

The area is rich in diverse terrain with islands and many waterways both narrow and wide. The environment is both challenging and suitable for the types of collaborative scenarios WARA-PS is interested in. WARA-PS also has access to very high quality 3D models of the region that can be used for motion planning and in interfaces for command and control stations. Figure 2 offers a figurative depiction of different types of WARA-PS activity around the castle area.

### Structure of the paper

The outline of the remaining part of the paper is as follows. Section 2 gives an overview of the WARA-PS infrastructure and the core system architecture, including the delegation framework. Section 3 presents results from two research projects on collaborative autonomous aerial vehicles for search and rescue missions, while Section 4 considers GPS free localization and human interaction for autonomous marine Vessels. Section 5 presents some selected WARA-PS research experiments and demonstration. Finally Section 6 gives a summary of the paper and suggestions for future research activities within WARA-PS.

## WARA-PS infrastructure and core system architecture

**Fig. 3:**
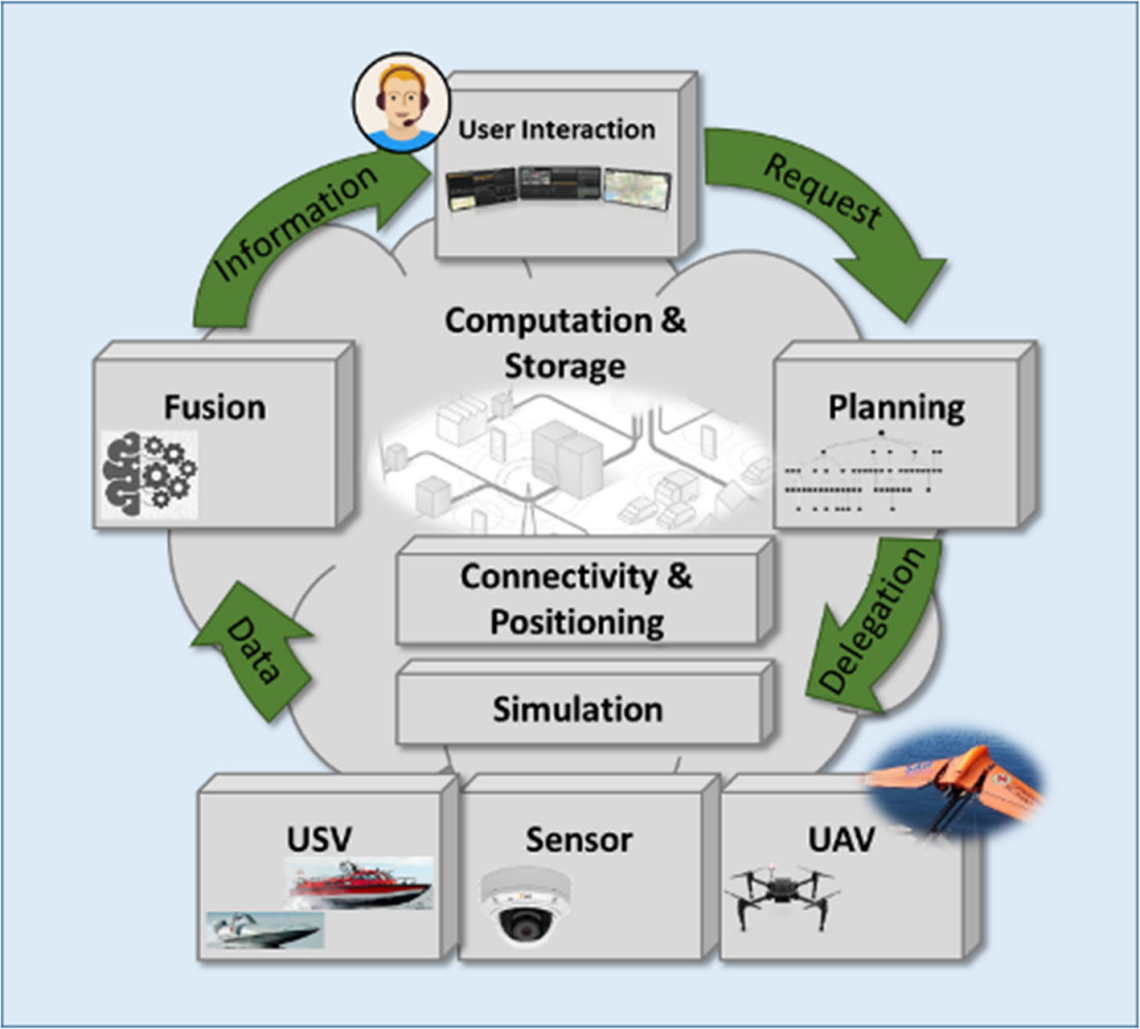
WARA-PS Infrastructure Overview

This section is intended to provide an overview of the WARA-PS Infrastructure and the Core System Architecture that supports the infrastructure. Figure 3 provides a high-level overview of the infrastructure. The basic control and data flow cycle involves many different and complex functionalities combining both hardware, software and robotic platforms. The infrastructure is intended to support highly complex public safety and security applications and scenarios. Support ranges from small scale two agent human/robotic scenarios to large scale multi-agent human/robotic scenarios. The infrastructure is intended to support varying levels of autonomy among robotic systems that dynamically change relative to the mission at hand. Much emphasis is placed on both collaborative robotics, human/robotic interaction, sensory perception and fusion, in addition to knowledge-level models derived from collected data and information that are used for decision support processes.

The basic information flow involves:

- multiple concurrent loops of user requests to multiple systems to accomplish mission goals (the Request arrow);
- various modes of trajectory, path and and collaborative task planning directed at both human and robotic agents where a delegation framework is often (but not always) used to generate executable specifications for participating human and robotic agents (the Delegation arrow);
- use of multiple robotic systems for air, land and sea to achieve mission goals. These missions often involve data collection activities used to provide different levels of situation awareness to participating human and robotic systems (the Data arrow);
- different streams of data at different levels of abstraction must continually be collected, stored, fused and visualized appropriately as information to be useful to human decision makers in ongoing missions (the Information arrow).

Any large scale public safety scenario naturally implies the use of robust computation and storage facilities in addition to communication networks operating at both real-time and soft real-time scales in order to ensure timely delivery of data and information to the right agent. An example of this would be real-time awareness of the positions of all participating agents in a scenario. Additionally, simulation tools are important for both user interaction and pre-mission and post-mission analysis. These functionalities are also part of the WARA-PS infrastructure.

This high-level perspective of WARA-PS frames the ambition and challenges involved in developing state-of-art systems of systems to support public safety applications. In the following, focus will first be placed on a more detailed description of the Delegation framework that is used for generating and executing complex, multi-agent distributed plans and tasks specified as Task Specification Trees. Considering its importance in the overall infrastructure, Task Specification Trees and the Delegation framework will be described in some detail. A more detailed description of the larger WARA-PS core system architecture that supports the infrastructure will then be described.

### Task specification trees

One of the core concepts in multi-agent systems and collaborative robotics is the concept of a *task*. Many different proposals for specifying tasks exist in the literature [14–20], although the majority have focused on single-agent/robotic systems. The move from single-agent/robotic systems to multi-agent and collaborative robotic systems adds to the complexity of finding an appropriate task specification language. There are a number of desirable properties such a language should have. It should be declarative with a clear semantics so it is easily understandable. It should have a procedural correlate to its declarative counterpart, so it is easily implementable and efficiently executable in agent/robot systems. It should also be extendable and scalable, in the sense that the target applications include heterogeneous robotic systems and teams of teams. Additionally, it should allow for both the specification of robotic activity, but also human activity, since human/robotic interaction is part and parcel of any public safety or emergency rescue scenario. One should also be able to specify tasks at any level of abstraction, from low-level reactive control activities to high-level deliberative activities. Finally, the language should allow for the parameterization of tasks and for the extension of tasks during runtime, in addition to being amenable to specifying shared tasks among multiple agent/robotic systems.

In previous work, a task specification language, called the Task Specification Tree (TST) Language has been proposed [21, 22] and used in the development of a framework for collaborative robotic systems [23, 24]. TSTs are intended to be used for both single-agent/robotic systems in addition to multi-agent/robotic systems. The TST framework has been continually developed and empirically tested in several field-robotic scenarios [25–29]. The framework currently plays a central role in the WARA-PS core system architecture and has been field-tested in this context using multiple heterogeneous robotic systems with human interaction.

**Fig. 4:**
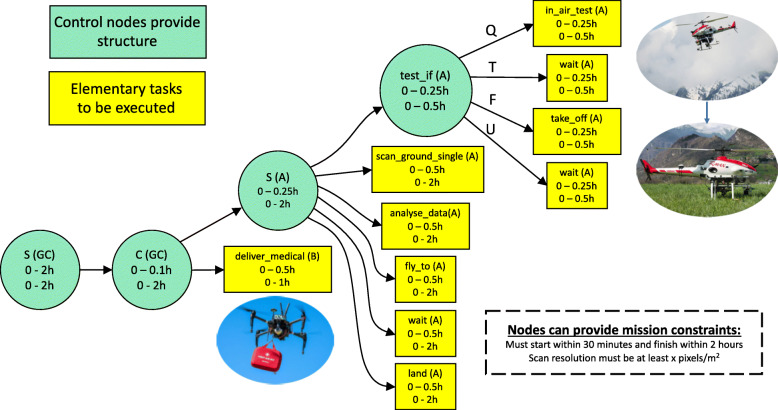
Collaborative Mission using three agents: (GC)– Ground Controller, (B) – DJI Matrice 100, and (A) – A Yamaha RMAX Helicopter

The essence of a task includes a set of elementary actions specified at a level where they are executable and a set of control structures that partially order the set of elementary actions. In collaborative scenarios, it is assumed that each robotic team member has *published* a set of elementary actions that can be used by the team in the formation of collaborative missions. TSTs are structured as trees, where leaf nodes consist of elementary actions and internal nodes consist of control structures. The following example, shown in Fig. 4, depicts a typical TST used in a collaborative mission consisting of three agents.

Each node in a TST is allocated to an agent/robot via a delegation process that is described in Section 2.2. In this example, a ground controller (GC) has responsibility for a mission in which two UAVs, a DJI Matrice 100 (Agent B), and a Yamaha RMAX (Agent A) are to concurrently execute two sub-tasks. The DJI is to deliver a medical supply kit to a pre-specified location and the RMAX is to execute a scanning operation in a pre-specified region. Before doing this, the RMAX checks whether it is in the air already or not. If not, it will take-off and then proceed with the mission. If it is in the air, it will simply proceed with the mission. This pre-check is specified using a test-if control node in which the RMAX is first queried and then the next task is determined by whether the query returns true, false, or unknown. Two other control nodes are used in this TST, a sequence node (S) and a concurrent node (C).

Constraint solving mechanisms are built into the framework where different types of constraints can be specified per node. In the example, each node has temporal constraints specifying lower and upper bounds for start and end times. In order for a TST to be valid and executable, these constraint problems must be solved. The constraint solving mechanism is described in more detail in Section 2.2.

The TST framework offers a rich set of node types that can be classified as follows:

- *Control Nodes* – (S) The Sequence node is a standard sequence construct; (C) The Concurrent node is a standard concurrency construct; (Test-If) The Test-If node is a general branching construct; (Sel) The Select node tests it’s children until one succeeds or all are checked; (Loop) The Loop node is a general loop construct where a sub-tree is repeatedly executed indeterminately until a termination condition occurs; (Monitor) The Monitor node sets up one or more temporal logic formulas expressing conditions to be incrementally evaluated over a sequence of states. Subtasks can be triggered if the formula becomes definitely true or definitely false, which is useful for execution monitoring [30] and failure recovery; (Try) The Try node corresponds to a try-catch-throw construct provided in many programming languages. It is useful for catching contingent problems and acting on them.
- *Interaction Nodes* – (Do) The Do node specifies a task for a human agent to fulfill, (Appr) The Approval node is used when a human operator must approve some data or return some new data that would await approval; (Query) The Query node offers a broad means of asking questions to either robot agents or human agents. The means of communication may be multi-modal and include visual or speech interfaces. (Goal) The Goal node contains an explicit high-level goal to be achieved and its execution allows an automated planner internal to an agent system to generate a plan which is then translated into a TST and spliced in the parent tree as a sub-tree to be achieved.
- *Built-in Elementary Action Nodes* – These nodes depend on the available platform types. Each node type may be supported by one or more platform types; for example, FlyTo may be supported by multiple aerial platforms. Two additional types were provided in the example, deliver_medical, and scan_ground_single.

### Delegation framework

**Fig. 5:**
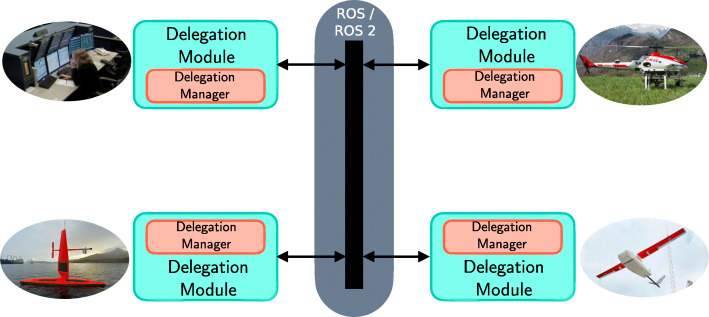
In a multi-agent system, the distributed task generation and execution network can be viewed as a dynamic graph that can grow and shrink as agent members enter and exit a particular mission in an operational environment

One of the backbones of the WARA-PS core architecture is a delegation framework [23, 24, 27, 31] that is used for generating and executing complex, multi-agent distributed plans and tasks. The tasks are specified using TSTs. One can abstract the actual delegation process as a dynamic graph where each team member is a node that participates in a delegation process. Figure 5 depicts this abstraction where the middleware solution used is ROS/ROS2 (Robot Operating System).

Given a high-level mission specification, provided by a member of the team, the purpose of the delegation framework is to dynamically instantiate an existing TST template in an agent’s TST library or dynamically generate a TST to achieve the mission specification. The resulting task specification often involves the use of a subset of members of the team whether it is instantiated statically or generated dynamically. The task specification is generated recursively through a process where participa- ting team members agree to do a part of the mission if they have the required resources and are able to commit to doing that part of the task specification. Each team member has the ability to broadcast for help in achieving sub-tasks associated with the larger mission specification. If successful, the net result of the process is the generation of a task specification tree where different parts of the specification are allocated to appropriate members of the team. An example of such a mission has been shown in Section 2.1.

As mentioned previously, it is assumed that each agent publicly commits to a set of elementary node/action types that can be used in the collaborative delegation process. In the case of UAVs, examples would be actions such as FlyTo, TakeOff, or Land. Elementary actions may also encapsulate more complex activities such as scanning of a region or delivery of a medical kit. While such activities are internally complex, and may in fact be expanded into composite actions, they are still elementary from the external point of view and can be used by the team to generate more complex task specifications collectively.

**Fig. 6:**
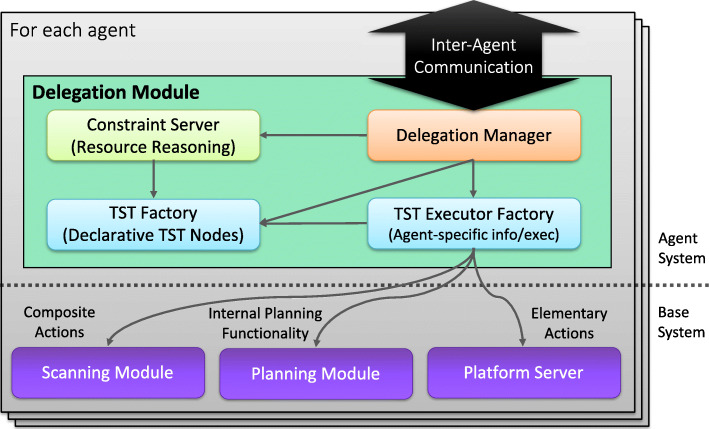
*Delegation Module* associated with each collaborative agent on a team

In the delegation framework, each member of a collaborative team is assumed to have a *Delegation Module* associated with it. An agent’s *Delegation Module* contains a Delegation Manager that manages the external interactions with other agents on the team, in addition to internally managing the generation and execution of composite tasks [22]. Figure 6 provides a high-level characterization of the internal architecture of a *Delegation Module*.

Each *Delegation Module* as shown in Fig. 6 consists of four conceptual components:

- *Delegation Manager* - It provides inter-agent communication to other members of the team during the delegation process. Internally, it accesses the TST Factory to generate TST nodes during the TST generation phase and the TST Executor factory to execute TSTs during the execution phase.
- *TST Factory* - It has the ability to generate TST nodes and TST sub-trees during the TST generation phase in the delegation process.
- *TST Executor Factory* - Associated with each elementary or composite action publicly declared by an agent, is a platform dependent executor that interfaces to an agents internal functionality. The TST executor factory is responsible for interfacing to and managing the execution of executors associated with elementary or composite actions for a specific platform. If a TST node is a goal node type, the TST executor also has the possibility to interface with an automated planner associated with a platform to generate a sub-tree from the planner that can then be used by the TST factory.
- *Constraint Server* - TST nodes can contain constraints that are inherited as the delegation process progresses. In order for an agent to answer the question “*can I do this?*” when it receives a request from another agent, it autonomously sets up a constraint problem and checks the problem for consistency, possibly returning specific variable bindings. The constraint server handles this part of the generation process. For instance, constraints can be temporal, resource based, or associated with sensor capability.

#### The delegation process

An initial Task Specification Tree, generated through templates or through automated planning techniques, can be viewed as a *goal request* representing a composite mission to be performed. In the example in Section 2.1, the TST has already gone through a delegation process and agents have been successfully allocated to nodes. For discussion, assume that nodes in the TST have not yet been allocated and the TST template is a goal request TST. It is therefore sent from the Ground Controller (GC) user interface to the GC’s local Delegation Module, which initiates a distributed delegation process where agents interact through their delegation modules. This process implements the abstract Delegate(*A, B, Task, Context*) speech act [31], where agent *A* wants to delegate *Task* to agent *B* given a *Context* specified as a set of constraints, through an interaction protocol with two phases [23].

In the first phase, tasks (TSTs) are provisionally allocated to agents capable of performing them while satisfying all mission constraints. In the second phase, the task allocation and a corresponding constraint solution can be presented to the operator, who can accept or reject it. If accepted, the participating agents are asked for a final commitment to the mission, which can then be started. Communication interaction between agents during a delegation process is achieved through the use of the FIPA Agent Communication Language [32] and FIPA Contract Net Interaction Protocol [33] based on Speech Acts.

The root node of a TST is always a control node and can be handled by any agent. For simplicity, we will assume this is delegated to the agent initiating the delegation process. In the case of the example, this is the Ground Control operator (GC). The interaction protocol therefore begins by sending a CALL-FOR-PROPOSAL speech act to this agent [32], indicating the task to be delegated together with the constraint context. From the contractor’s point of view, the remainder of the first phase of the protocol can be characterized using the DELEGATE-FIRST-PHASE procedure below^1^.


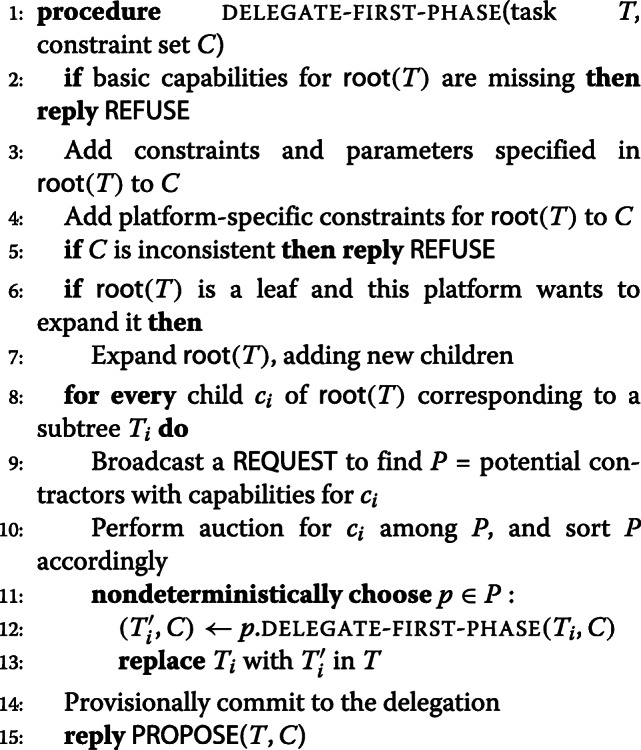


[Line 2] An agent can only be allocated a tree *T* if it can execute its root. The agent therefore begins by verifying that it has the necessary fundamental capabilities. If capabilities are missing, the agent immediately responds using a REFUSE speech act.

[Line 3-5] The agent must also verify that it can execute the task given the specified parameters and constraints. If the resulting constraint set is inconsistent, the agent cannot accept the delegation and must reply REFUSE.

[Line 6-8] Otherwise delegation *may* be possible, contingent on the successful delegation of all children. These children may already exist or may be generated dynamically through a potentially platform-specific expansion procedure provided by the TST Executor Factory.

[Line 9] For each child *c*_*i*_, associated with a subtree *T*_*i*_, a REQUEST for potential participants will be broadcast. This request is accompanied by a specification of the required capabilities for *c*_*i*_, which allows replies (sent as INFORM speech acts) to be filtered.

[Line 10] An auction process is then initiated where each potential contractor is REQUESTed to bid for the task in question. Each bid is also returned through an INFORM speech act.

[Line 11] Bids are used to prioritize potential contractors, but backtracking may be needed if a choice that is good for one part of the TST has negative consequences for other parts of the tree. For brevity we describe this backtracking using the standard notion of non-deterministic choice, where each such choice point is in fact a point to which the algorithm can backtrack in case of future failures.

[Line 12-15] When a child has been provisionally delegated, its subtree may contain expanded nodes, and the nodes of the resulting tree are associated with execution constraints defined by the contractor(s) that were allocated parts of this tree. The expanded tree and updated set of constraints are returned in line 15 and the corresponding values returned from a recursive delegation call are handled in lines 12–13. When the first phase of delegation succeeds (line 14), the platform also *provisionally* commits to the delegated task before it PROPOSEs a solution to the caller. The commitment is provisional both because one may backtrack over the commitment and because no delegation is final until the original delegator has received a proposed solution and accepted it. This allows a ground operator to determine whether a mission instantiation is acceptable or whether an alternative needs to be sought.

##### Second phase.

If the mission is accepted, an ACCEPT speech act is distributed to all callers, also specifying a concrete constraint solution to be used during execution. Otherwise, an REJECT speech act is distributed.

### The WARA-PS core system architecture

Developing a core system architecture for WARA-PS activity is a challenging endeavor with many different levels of complexity. Ideally, one would like to use the template depicted in Fig. 5 and described in Section 2.2 to allow heterogeneous robotic and human agents to homogeneously collaborate and interact through a common software template such as the delegation module. To some extent, this is an option since a custom NUC hardware module with the requisite delegation and other software modules has been packaged as an out-of-the-box system to be easily integrated with any robotic or ground control station that supports Linux and ROS. This hardware module is described in Section 3. This solution has been used for collaborative robotics research with teams of humans and DJI quadrotor systems within the IDA-AIICS research group which is one of the participants in WARA-PS.

The requirements for a collaborative robotics architecture are much more complex due to the variety of participants and the different uses of WARA-PS. The primary goal is to provide a space for research and development between academic groups from diverse disciplines and companies with diverse activities. Each of the partners has its own unique requirements that are not easily accommodated in one uniform choice for all aspects of the shared architecture. Consequently, the WARA-PS architecture embraces and supports the diversity of requirements in the construction of a sophisticated multi-user system that tackles this diversity head-on.

One of the first important choices to make is in determining what middleware solution to use for the architecture. In general, *middleware* is software that allows other software and applications to communicate and interact seamlessly in a distributed system setting. This of course is an essential component in the collaborative robotics setting targeted by WARA-PS. Middleware solutions can be highly generic, ranging from solutions such as CORBA [34] (Common Object Resource Broker), a service-oriented architecture where all entities are viewed as objects that can share services, to more application specific solutions, targeting specific entities such as robotic systems. ROS [35] (Robot Operating System) is an example of the latter.

ROS “provides libraries and tools to help software developers create robot applications. It provides hardware abstraction, device drivers, libraries, visualizers, message-passing, package management, and more” [35]. ROS is highly popular in the academic community among robotics research groups and is becoming increasingly popular in industry (e.g. ROS-Industrial [36]). It not only offers middleware solutions though the use of topics (publish/subscribe messaging transport) and remote procedure calls (services), but has a large user group contributing useful software that can be shared across the community. ROS was originally single-robot centric, but this is now changing with the advent of ROS2.

One of the choices for middleware in WARA-PS is ROS/ROS2. The delegation framework described in Section 2.2, uses ROS/ROS2 as its primary middleware choice. ROS is not as prevalent among participating industrial partners in WARA-PS. Early experimentation was done with a number of company in-house solutions, but since these are proprietary, they were found to be less robust in terms of the general requirements involved in WARA-PS. One alternative middleware solution one has recently gravitated toward is a more generic software entity oriented solution, MQTT [37].

MQTT “is an OASIS standard messaging protocol for the Internet of Things (IoT). It is designed as an extremely lightweight publish/subscribe messaging transport that is ideal for connecting remote devices with a small code footprint and minimal network bandwidth” [37]. MQTT is very popular in industry due to its standardization and light footprint. Due to its use among some of the WARA-PS industrial partners, a choice was made to use both ROS/ROS2 and MQTT in an integrated manner as the middleware backbone for the WARA-PS architecture. One of the advantages of MQTT is that it is supported on all platforms without a specific requirement for ROS or Linux. Additionally, it is straightforward to create bridges between MQTT and ROS topics which makes communications transparent.

Another architectural choice that has been made is to diversify the concept of *agent* to meet the requirements of the different partners in WARA-PS. Robotic systems that integrate the full delegation framework with its delegation module and manager are called *delegation agents*. For teams of delegation agents, very powerful modes of collaboration are offered seamlessly, dynamically and autonomously, due to the nature of the functionality included in the delegation framework. Delegation agents are currently the most sophisticated agent type in the architecture lying at the top of a conceptual agent stack.

There are different levels of complexity in the use of the WARA-PS infrastructure that have to be taken into account. For some users, data collection is the dominant target for use of the arena functionality, so packaging sensors as delegation agents could be considered overkill. Additionally, participating companies with proprietary systems such as Kockum’s Piraya marine surface vehicle might want to bypass the delegation framework in some experimentation and call tasks directly through dedicated command and control interfaces. Others might want to do this but also take advantage of the features inherent in viewing tasks as task specification trees (TSTs). Consequently, a dynamic hierarchy of agent types has been defined to accommodate the diverse needs of the different arena users. These agents types are all compatible with each other and independent of each other. Additionally, the hierarchy is easily extendable.

Currently there are four different types of agents accommodated with the option of defining additional agents relative to need. Each agent level is essentially defined by support for a particular JSON based API/protocol (one for each level). Supporting a particular JSON based API/protocol is what defines a particular agent/agent level. This implies that the agent/agent levels are independent capabilities. Several of these agent types can be combined hierarchically:

- *Sensor Agent* – Sensor agents are the most basic type of agent. A sensor agent provides one or more streams of data as output in addition to a heartbeat signal. Data streams can also be abstract and provide meta-data about an agent too. Communication is essentially in one direction only. Sensor agents can communicate through an MQTT broker and MQTT topics or through ROS and ROS topics. A configurable message replication mechanism is provided through both MQTT and ROS topics. For example, filtered MQTT based streams can be passed onto ROS topics and vice-versa. An example of a sensor agent would be a camera on a robotic system or a static camera on land.
- *Direct Execution Agent* – a Direct Execution agent supports all functionality a sensor agent supports in addition to being able to directly execute a task, one task at a time. It can be queried about supported tasks and provide current state of a running task. A task can be started, paused or terminated. Communication with a Direct Execution agent is compatible with both MQTT and ROS. An example of usage would be a proprietary robotic system where one would like limited participation in a collaborative mission by commanding tasks from a ground station, but all internals as to execution of the task, etc., are hidden from the team.
- *TST Execution Agent* – A TST execution agent supports all functionality A Direct Execution agent supports, in addition to supporting execution of pre-assigned TSTs. Communication with a TST Execution agent is compatible with both MQTT and ROS. This particular type of agent is useful in the context of human users interacting with robots by commanding their TST suites through user interfaces. Here there is not a requirement for ROS but the execution environment for TSTs is accessible.
- *Delegation Agent* – A Delegation agent is supported by the full delegation framework, where TSTs can be dynamically generated and executed, the agent can participate in the delegation process, and full support for constraint handling is provided. Communication with a Delegation agent is compatible with both MQTT and ROS.

Each of these different types of agents and their combinations are widely used in the WARA-PS architecture. One particularly interesting example of this is the modular extension of a Direct Execution agent into a TST Execution Agent. This is depicted in the diagram (below) as the “Piraya agent”. Kockum’s Piraya marine surface vehicle is originally defined as a Direct Execution Agent. Direct execution agents can support internal tasks that are declaratively specified in essentially the same way as TST nodes. From this specification, one can automatically generate code for TST executors that uses a standard direct execution interface defined for the WARA-PS collaborative architecture. Given that code for TST executors is automatically generated, one can then create a *virtual* TST Execution agent *associated* with the piraya. This TST agent does not have to be part of the physical piraya architecture, but can reside anywhere in the larger collaborative system architecture, for example on a ground station or even in the WARA-PS Cloud. The piraya can then be accessed as a TST-based system through the virtual piraya TST Execution agent. There are many variations of this basic idea of combining agent types that have proved to be very useful.

**Fig. 7:**
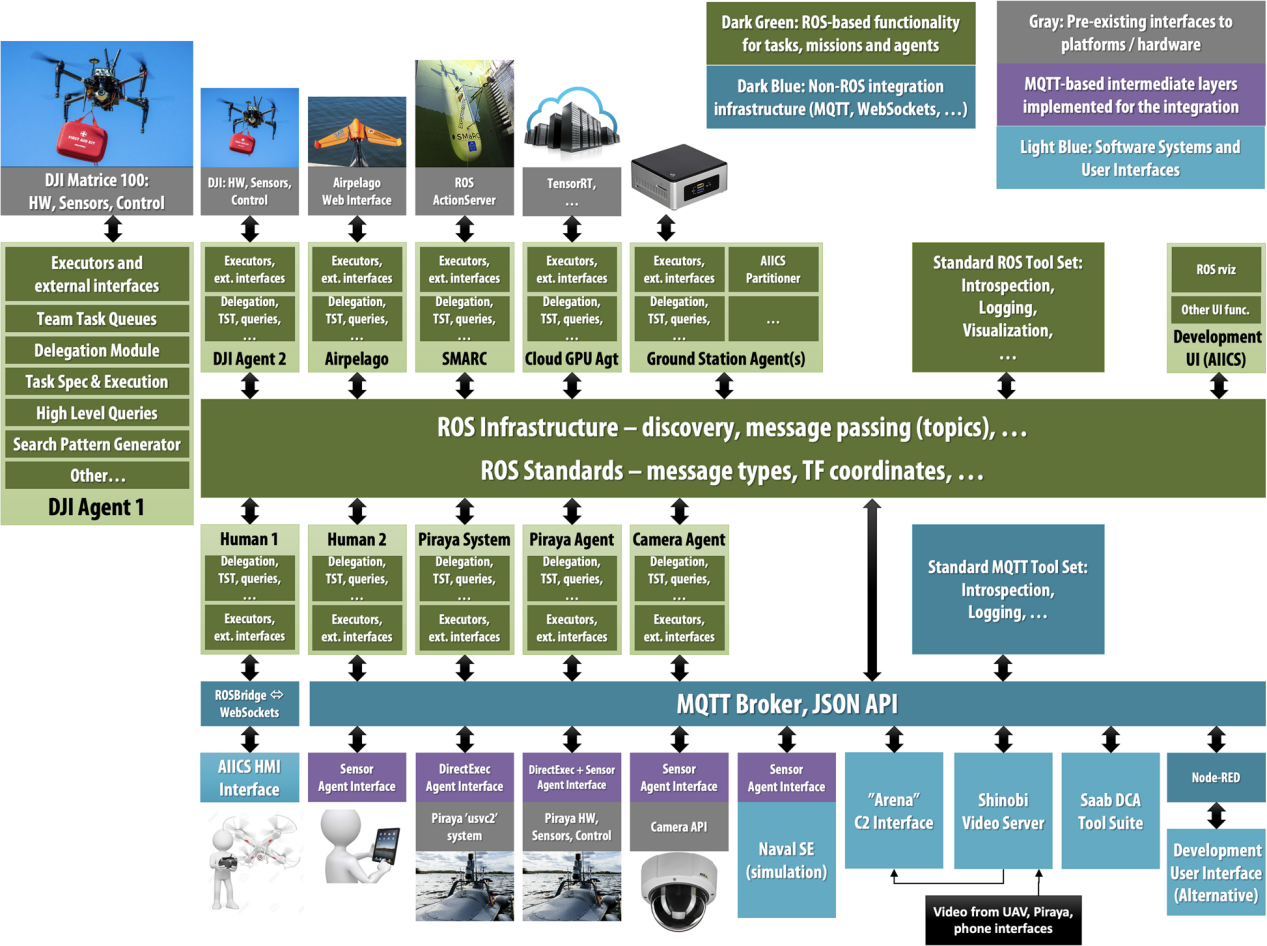
A Schematic of the WARA Public Safety Collaborative Systems Architecture for research and experimentation

A schematic of the WARA-PS arena architecture is provided in Fig. 7.

Note that the architecture supports not only robotic systems from different academic and industrial partners, but also offers distributed ground station support, distributed database storage support and Cloud-based storage and computational support. Generally, these non-agent entities are wrapped as agent types to have full access to participating systems in the arena and vice-versa. For example Kockums AB is interested in experimenting with one of their products, Navel SE, a maritime simulation and real-time tracking system that visualizes maritime vehicles at sea and offers information about them. This system is currently wrapped as a Sensor agent, where positions of all maritime vehicles in the WARA-PS operational environment can be broadcast to other robotic systems and ground stations.

The WARA-PS core system architecture is under continual development and expansion and has been used successfully in many complex multi-robotic, multi-human scenarios. Many of the components and functionalities are being provided by both academic and industrial partners. The architecture has been setup to ensure the ability to continually add new functionality, platforms and features in a modular and efficient way.

For example, a data collection and analytics (DCA) tool suite provided by Saab (lower right corner of Fig. 7) puts data into the context of time and space where it can be visualized and investigated. The tool suite contains applications for data analytics that can be used both on- and off-line. By storing data in these dimensions (x, y, z, time), it enables a number of interesting features such as searching for objects in an area over a given timespan. The tool suite is a sensor and application independent product that can be run as a stand-alone product or be easily integrated into other applications. The main functionalities of the product includes:

1. Big Data platform – Capable of storing and retrieving massive amounts of data at a very high pace.
2. High Performance Computation Platform – For inference and training of Artificial Intelligence (AI)algorithms.
3. Micro-Service Platform – Ensures scalability, enabling easy add-on functionality and low-cost integration.

**Fig. 8:**
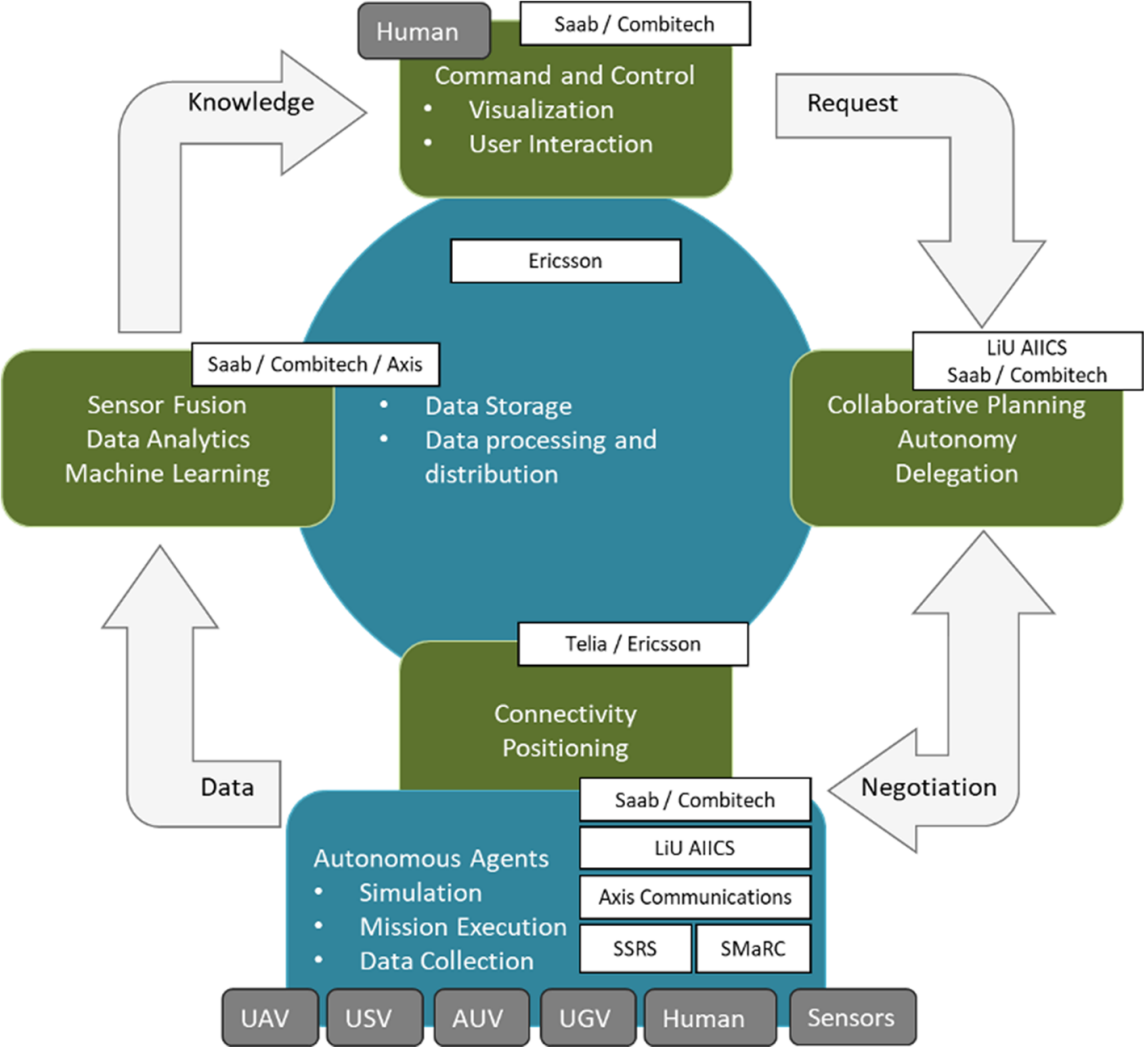
Company participation and integration in the WARA-PS infrastructure

In Fig. 3 a conceptual description of the WARA-PS infrastructure has been presented with a discussion of data, decision and control flow. The participating companies in WARA-PS have major interest in many of the functionalities and processes associated with the infrastructure, not only in terms of research and development, but in providing products that can be integrated in the infrastructure and tested with complex use cases. Figure 8 emphasizes the deep integration of the participating companies in the many different parts of the infrastructure.

### The WARA-PS research portal

The WARA-PS Research Portal [38] is a web-based platform for sharing resources within WARA-PS. Accessing resources in the project is crucial in encouraging further development of the technologies and integration of systems. The aim of the WARA-PS Research Portal is to provide necessary resources and information about the infrastructure, platforms and their use in a straightforward and efficient way. The portal also provides a virtual meeting place containing ROS development and simulation environments where researchers can access both interfaces and study use examples packaged in tutorials and self-study courses.

## Collaborative autonomous drones (UAV) experimentation

WARA-PS provides a challenging environment for research with collaborative UAVs. A great deal of experimentation has already been done in the Gränsö area using dedicated drone teams. This section describes the platforms used and some of the field robotic experimentation done.

### Aerial vehicle platforms used in WARA-PS

#### DJI matrice 100/600 platforms

WARA-PS has access to a fleet of 4 enhanced DJI Matrice 100’s and one enhanced DJI Matrice 600 used for research and experimentation. These systems have been equipped with various types of sensors in addition to an onboard computer system.

**Fig. 9:**
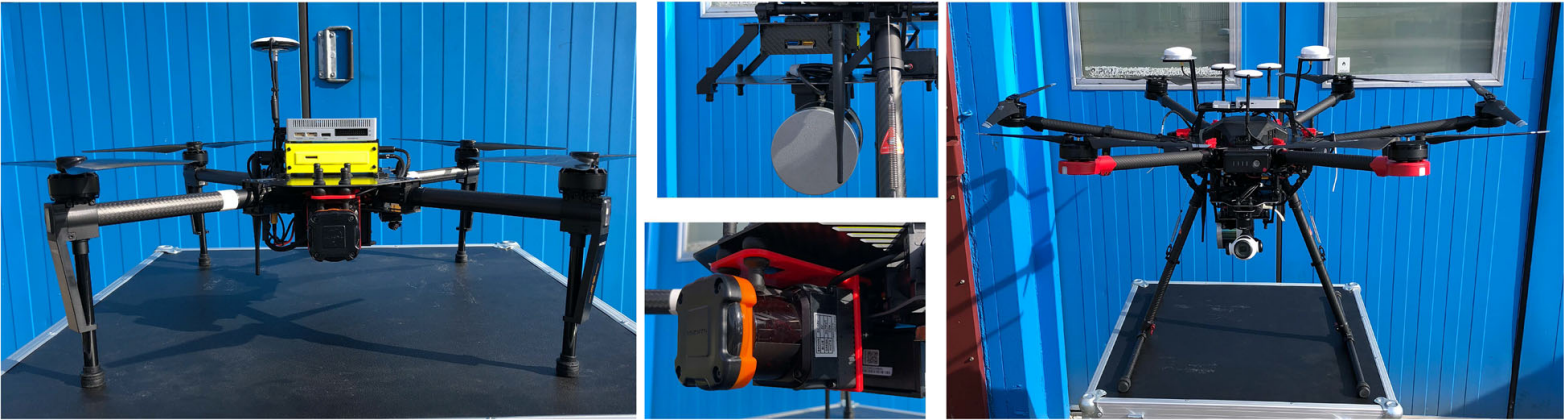
Experimental platforms: DJI Matrice 100 equipped with a Hokuyo UTM-30LX LIDAR sensor (left), DJI Matrice 600 Pro equipped with a Velodyne Puck LIDAR sensor (right)

The first, shown on the left of Fig. 9, is a modified DJI Matrice 100. It has a maximum takeoff weight of 3.6kg and 1.2kg of payload capacity. The platform measures 100cm between propeller tips. It can fly with speeds up to 22m/s and has a maximum flight endurance of 22 minutes. The platform is equipped with a Hokuyo UTM-30LX LIDAR, which is a single scan device with a guaranteed range of 30m (60m maximum).

The second type of platform, shown on the right in Fig. 9, is a modified DJI Matrice 600 Pro. It has a 15.1kg maximum takeoff weight, 6kg of payload capacity, maximum flight speed of 18m/s, and 35 minutes of flight time using 5.5kg of payload. It measures 167cm between propeller tips. The GPS system on-board uses a Real-Time Kinematic (RTK) positioning technique to deliver centimeter accuracy measurements. This particular platform is equipped with a Velodyne PUCK LIDAR sensor, which has an effective range of 100m and uses 16 scan channels. An LIDAR mounting mechanism developed and deployed on the DJI Matrice 600 Pro allows for choosing the sensor orientation depending on the applications or missions at hand.

Both platforms are equipped with the same type of onboard computer system. It is an Intel NUC Kaby Lake i7-7567U CPU platform in a custom enclosure equipped with 16GB of RAM and 500GB SSD of storage. The computer systems interface with the platforms and run among other things, the software modules associated with the *Delegation Module* described previously in Section 2. This setup allows for a modular extension of team members by integrating the Intel NUC module with any new robotic team member. The communication with the ground station for both platforms is realised using 5GHz WiFi connections.

**Fig. 10:**
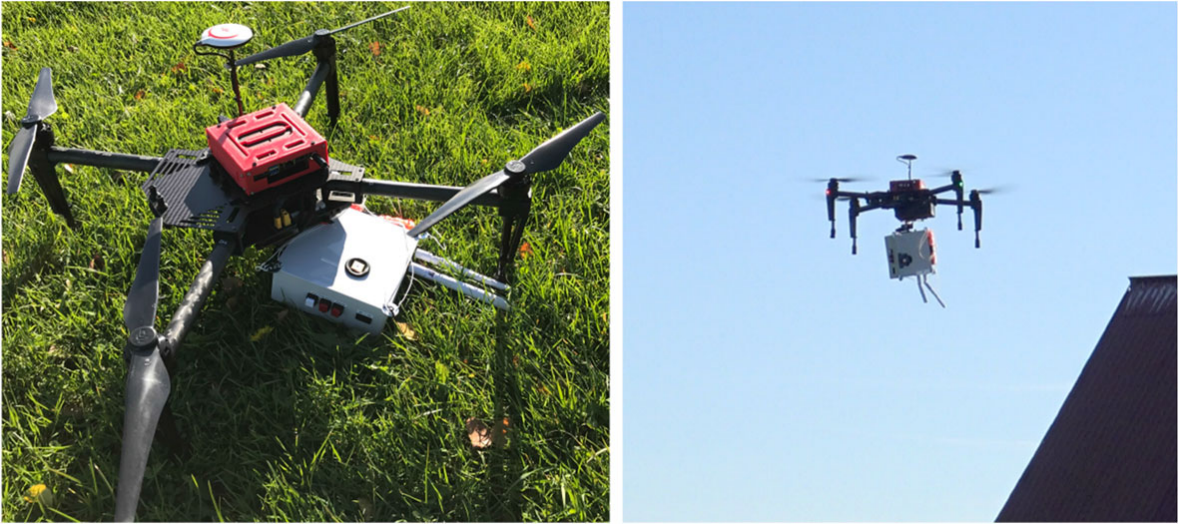
The DJI Matrice 100 system with a CommKit attached

Some of the systems have also been extended with an autonomous delivery system, where packages such as emergency medical aid or communications can be autonomously deployed in operational areas. Figure 10 shows one of the DJI 100 platforms carrying a CommKit system which is deployed autonomously.

The central component of the CommKit system is a WiFi Access Point (AP) which is used for the creation of ad hoc wireless networks between multiple CommKits delivered by the DJI 100s.

#### SSRS fixed wing platform

**Fig. 11:**
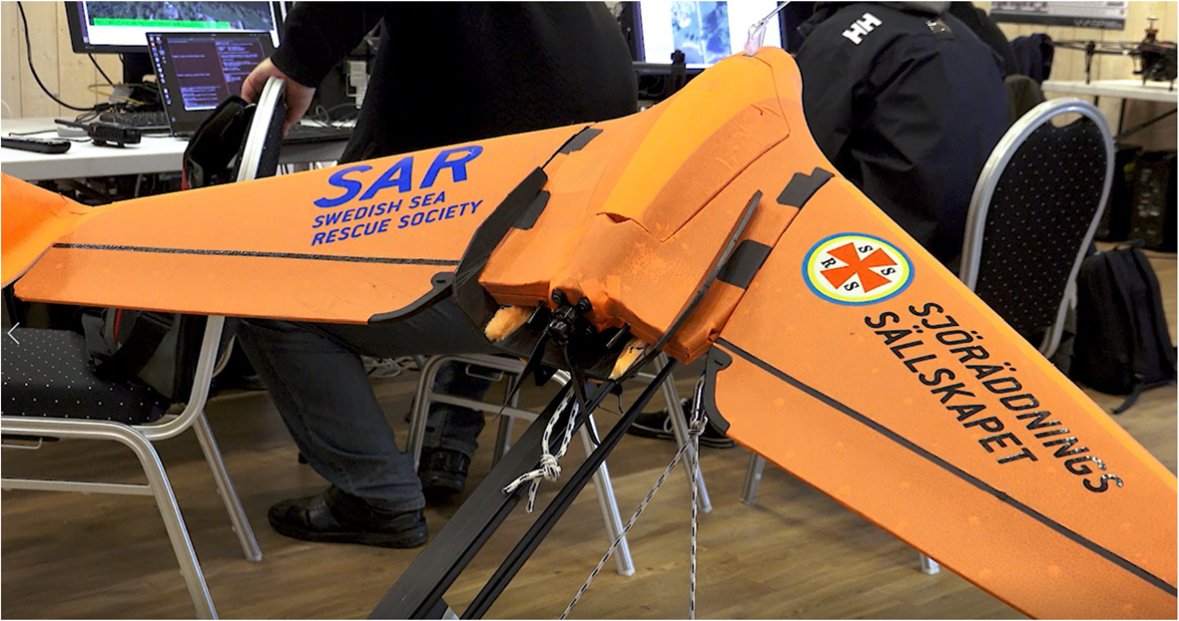
SSRS fixed wing drone developed in cooperation with Airpelago

Airpelago [39] is a Swedish company that develops and provides software for drone applications, in particular multi-drone applications. One of their first case studies involved development of a fixed-wing drone for sea rescue in cooperation with SSRS [13], the Swedish Sea Rescue Society. The SSRS fixed wing platform is depicted below in Fig. 11 and has been used in several of the WARA-PS scenarios. It has been used primarily for circulating over and monitoring the site of accidents or rescue events, providing live streaming of video footage of particular regions of interest. The idea is to improve situation awareness quickly by providing a first overview for better estimation of the magnitude of an accident. The system contains a launcher and planning software as well as AIS integration. It is designed with the intention of flying beyond line-of-sight.

### Selected research with aerial vehicles

#### Autonomous landing and deployment of aerial vehicles from surface vehicles

The automatic deployment and landings of Unmanned Aerial Vehicles (UAVs) on Unmanned Sea Vessels (USVs) is a crucial feature when operating a search-and-rescue mission in remote areas at sea. The limited battery capacity of drones gives them a limited flight range from their take-off point. Before the battery runs out, a USV must be located, followed, and landed on. Since the USV might be driving towards some place of interest, the landing must be possible also on a moving landing platform.

The autonomous landing problem is challenging for many reasons. First, the control requires the coordination of heterogeneous vehicles under the influence of disturbances from wind and waves. Because both vehicles are autonomous and neither of them is following a pre-specified path, they need to communicate and negotiate their future control actions to generate a safe landing trajectory. Second, the vehicles need very accurate estimations of their relative positions, as well as of the velocities and headings of the other vehicle. This is achieved both by communicating sensor information and by using sensors such as cameras to measure the other vehicle.

To generate feasible rendezvous trajectories for the vehicles, we use Model Predictive Control (MPC). MPC is an optimization based control framework in which an optimal state trajectory ***x***^⋆^ and corresponding control inputs ***u***^⋆^ are solved for in every sampling time. Traditional MPC has a control horizon *N* over which the control inputs are optimized, giving a total look-ahead time of *N*·*dt*, where *dt* is the sampling time. MPC is particularly suitable for solving constrained control problems, since the constraints can be taken into account directly in the optimization problem. The work in [40–42] develops an MPC for the autonomous landing problem between a quadcoper UAV and a USV. The objective function is here expressed as the sum of weighted squared distance to the rendezvous state $\bar {x}$ and input $\bar {u}$

$$ J(x_{0}, \boldsymbol u) = \sum_{i=0}^{N-1} \left\lVert x_{i} - \bar{x}\right\rVert^{2}_{Q} + \left\lVert u_{i} - \bar{u}\right\rVert^{2}_{R} + \left\lVert x_{N} - \bar{x}\right\rVert^{2}_{Q_{f}}. $$

**Fig. 12:**
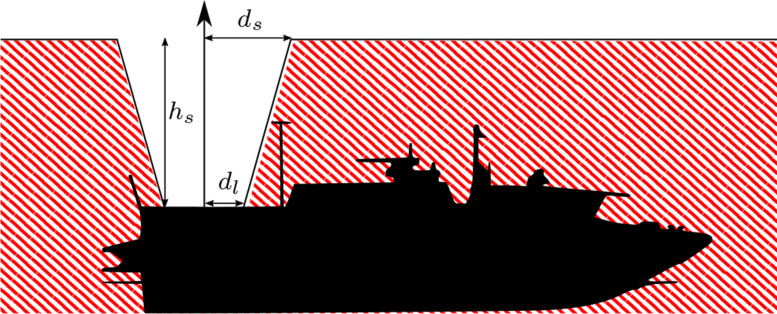
The red region is considered a dangerous area for the quadcopter to enter, because of the protruding parts and potential closeness to human operators. The MPC ensures the avoidance of the region

**Fig. 13:**
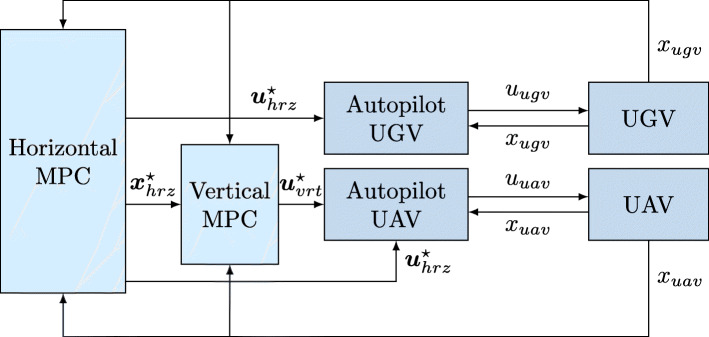
The separated MPC architecture. The optimal solutions from the MPC are sent to low-level autopilots that compute the actuator inputs

Spatial safety-based constraints are added for avoiding masts, antennas, and other protruding boat parts during the landing, see Fig. 12. Because the constraint is nonconvex, a mixed-integer program has to be solved if it is included in the MPC, which is known to be intractable for long horizon problems. To speed up the solution time, the problem is made convex by implementing the controller as separated into two parts – one representing the horizontal dynamics, and the other representing the vertical dynamics. The optimal trajectory resulting from the horizontal MPC is then used as an input to the vertical MPC, which plans a safe descent trajectory. This architecture is illustrated in Fig. 13.

In [43, 44], the results are extended to the use of Variable-Horizon MPC (VH-MPC). This MPC framework lets the horizon be an integer variable of the optimization problem, such that the remaining maneuver time can be weighed against control inputs and other signals of interest in the objective function. VH-MPC has many advantages over MPC for rendezvous type problems, such as an autonomous landing. First, the horizon can be extended to make the problem feasible with respect to the terminal constraint if the initial state is distant from the terminal state. This means that the entire maneuver can be planned and we do not have to constrain where we start the maneuver with respect to the relative distance. Second, when applying VH-MPC, the horizon can be included in the cost function, for example as

1 $$ {}J_{N}(x_{0}, \boldsymbol u) = \sum_{i=0}^{N-1} \left\lVert x_{i} - \bar{x}\right\rVert^{2}_{Q} + \left\lVert u_{i} - \bar{u}\right\rVert^{2}_{R} + \left\lVert x_{N} - \bar{x}\right\rVert^{2}_{Q_{f}}+ c\cdot N,  $$

where *c*≥0 is a scalar weight. If we now select the terminal set to be equal to a set around the rendezvous point, this means that the objective function indirectly penalizes the total maneuver time. This means that in (1), the weight in *Q* and *Q*_*f*_ corresponding to the distance to the rendezvous can be set to zero, resulting in more appropriate rendezvous trajectories. A further benefit of applying VH-MPC is that the horizon becomes shorter as the vehicles approach the landing. This means also that the optimization problem becomes computationally less expensive at the final part of the maneuver. This is significant because it is particularly important that we are able to solve the problem on time in this critical part of the maneuver.

The biggest challenge with implementing VH-MPC in practice is that several MPC problems of different horizons have to be solved within each sampling time. This can make the controller computationally intractable, in particular when the horizons are long. To mitigate this difficulty, [44] derives a computationally efficient algorithm for VH-MPC. First, the problem is separated into an inner and an outer problem, where the inner problem corresponds to a standard linear MPC problem of a specific horizon *N*

2 $$\begin{array}{*{20}l} {}\begin{array}{lll} \underset{\boldsymbol u}{\text{minimize}}\quad & J_{N}(x_{0}, \boldsymbol u) \\ \text{subject to}\quad & x_{0} = x(0) \\ &x_{k+1} = F x_{k} + G u_{k} & \text{for }k=0,1,\ldots,N-1, \\ &c_{k} \leq C x_{k} + {Du}_{k} \leq d_{k}& \text{for }k=0,1,\ldots,N-1, \\ & c_{N} \leq C_{N} \xi_{N} \leq d_{N}. \end{array} \end{array} $$

The outer problem now becomes that of solving the following integer optimization

3 $$\begin{array}{*{20}l} \begin{array}{lll} \underset{N^{0}}{\text{minimize}} \quad & J_{N^{0}}(x_{0}, \boldsymbol{u})\\ \text{subject to} \quad& (2)~\text{is feasible}. \end{array} \end{array} $$

**Fig. 14:**
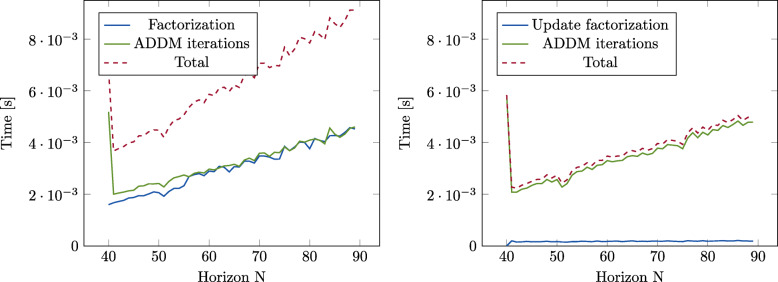
The time to solve one MPC problem for different horizons, using the standard OSQP algorithm (left) and out recursive factorization (right). Our method reduces the solve time by removing the need to refactorize the matrix when changing the horizon

The number of optimization problems that have to be solved in each sampling time in (3) can be reduced by selecting suitable terminal constraints and costs. Still, the involved problems have to be solved very fast in order to reduce the total solve time. In order to do this, the main idea of our algorithm is to utilize the similarities between the optimization matrices corresponding to the different horizons in (2), by deriving a recursive factorization method that is easy to extend or truncate. The method is based on using OSQP [45], which is a state-of-the-art QP solver implementing the ADMM method. The solution of each QP corresponds to the factorization and iterative backsolving of the following matrix

4 $$\begin{array}{*{20}l} \left[\begin{array}{ll} P + I\sigma & A^{T} \\ A & -\rho^{-1} I \end{array}\right] \left[\begin{array}{ll} \tilde{x}^{k+1} \\ \tilde{\nu}^{k+1} \end{array}\right] = \left[\begin{array}{ll} \sigma x^{k} -q\\ z^{k} - \rho^{-1}y^{k} \end{array}\right], \end{array} $$

where $\tilde {x}$ is an auxiliary variable, $\tilde \nu $ is a dual variable, and *σ* and *ρ* are step-size parameters. *P* and *A* are the cost and constraint matrices, which will vary depending on the horizon. Instead of redoing the factorization of the KKT matrix in every iteration, we derive a forward recursion method using the Shur complement for the factorization. The final factorization of the KKT matrix can be written as

$$P_{0}^{T}{KP}_{0} = LDL^{T} $$

with matrices

$$\begin{array}{*{20}l} &L = {\left[\begin{array}{ccccc} L_{0} & \\ \bar{Y}_{01}^{T}L_{0} &L_{1} & \\ & \bar{Y}_{11}^{T}L_{1} & \ddots \\ & & \ddots & L_{2N}\\ & & & Y^{T}_{2N,1} L_{2N} & L_{2N{+}1} \end{array}\right]},~\\& D = {\left[\begin{array}{ccccc} D_{0} \\ & -D_{1} \\ &&\ddots \\ &&& D_{2N}\\ &&&& -D_{2N{+}1} \end{array}\right]}, \end{array} $$

and where *P*_0_ is a permutation matrix. The matrices can now be used to repeatedly solve equations on the form (4) for different horizons by extending or truncating the *L*,*D* and *P*_0_ matrices. This way, the cost of re-factorizing the problem for each new horizon is removed. This is illustrated for an example problem in Fig. 14.

The efficient update algorithm for the factorization, together with the smaller number of evaluated horizons result in a computationally tractable VH-MPC algorithm, which can find the optimal horizon and decrease it as the system approaches the terminal set. Experiments on the real system, as described in Section 5, are used to illustrate the real-time applicability of the algorithm as well as its effectiveness under disturbances.

#### Planning safe trajectories using machine-learned proximity constraints and informed aerial search for victims

When developing autonomy for robots to be deployed in public spaces, a common problem is how to deal with the uncertainty inherent in complex real world environments. In search and rescue applications there is uncertainty both on the mission level, e.g. in which areas one expects to find victims, but also many sources of uncertainty for individual robots navigating in a dynamic and chaotic disaster zone.

It is well-known that planning under uncertainty can be formalized as finding a policy *π*(**x**) that minimizes the future expected cost [46],

5 $$ \begin{aligned} & \underset{\pi(\boldsymbol{x})}{\text{arg\;min}} & & \mathbb{E}_{\boldsymbol{x}_{t:t+H}} \left[c(\tau_{t:t+H})\right], \\ \end{aligned}  $$

where *τ*_*t*:*t*+*H*_ is a future state-action trajectory through the environment and the cost function *c*(*τ*_*t*:*t*+*H*_) encodes task objectives. Unfortunately, uncertainty makes already challenging planning problems computationally intractable, as they now have to be solved under the expectation operator. For real-world robots we require approximations that i) can be solved in real-time, and ii) satisfy any application safety requirements.

Applications in aerial vehicles such as quadcopters are particularly challenging due to the limited on-board computing capacity, as well as the dangers that rotorcraft pose to people when they are used in populated public spaces. Our research in this area includes finding tractable and safe approximations to this problem with applications in search and rescue [47]. These include safely navigating among people when searching for victims or inspecting damage, as well as mission planning for informed aerial search of victims.

In safety critical applications such as autonomous robots navigating around people, it is helpful to include transparent notions of safety such as imposing an explicit constraint on the planning problem,

6 $$ \begin{aligned} & \underset{\pi(\boldsymbol{x})}{\text{arg\;min}} & & \mathbb{E}_{\boldsymbol{x}_{t:t+H}} \left[c(\tau_{t:t+H})\right] \\ & \text{subject to} & & \\ & & & \Pr(\boldsymbol{g}(\tau_{t:t+H}) \geq \boldsymbol{0}) > p.\\ \end{aligned}  $$

For example, the safety constraint *g*(.) in obstacle avoidance problems can be encoded as a minimum distance to obstacles. Due to the uncertainty inherent in the problem, for example from the motion of people and the robot itself, we use a statistical notion of safety that should be satisfied with a high probability *p*.

One line of research pursued is to learn deterministic approximations to the probabilistic problem in Eq. 6, where the probabilistic safety constraint is still guaranteed by a parameterized approximation ***g***_*θ*_(*τ*_*t*:*t*+*H*_) [48].

The resulting MPC problem with learned soft constraints via slack variables ***δ*** and safety parameters *θ* then becomes,

7 $$ \begin{aligned} & \underset{\tau_{t:t+H},\, \boldsymbol \delta}{\text{arg\;min}} & & c(\tau_{t:t+H,},\boldsymbol \delta) \\ & \text{subject to} & & \\ & & & \boldsymbol{x}_{i+1} = \boldsymbol{f}(\boldsymbol{x}_{i}, \boldsymbol{u}_{i}),\, \forall i,\\ & & & \boldsymbol{x}_{t} = \boldsymbol{\hat{x}}(t),\\ & & & \boldsymbol{g}_{\theta}(\tau_{t:t+H}) \geq \boldsymbol{0}\,-\,\boldsymbol \delta,\\ & & & \boldsymbol \delta \geq \boldsymbol{0}.\\ \end{aligned}  $$

This is a safe *determinized* approximation to the original problem with uncertainty. Since this is now a conventional deterministic *trajectory* optimization problem, safe trajectories can be computed by off-the-shelf MPC solvers such as FORCES [49] or ACADO [50].

To learn safe constraint approximations we reframe the problem as a policy search where the policy *π*_*θ*_(***x***), is the MPC program in Eq. 7 parameterized by its safety constraint parameters *θ*. By leveraging recent advances in constrained Bayesian optimization [51, 52], such a policy can be automatically optimized to satisfy probabilistic safety constraints Pr(***g***(*τ*_*t*:*t*+*H*_) ≥***0***) >*p* with high probability using either simulations or real-world testing.

**Fig. 15:**
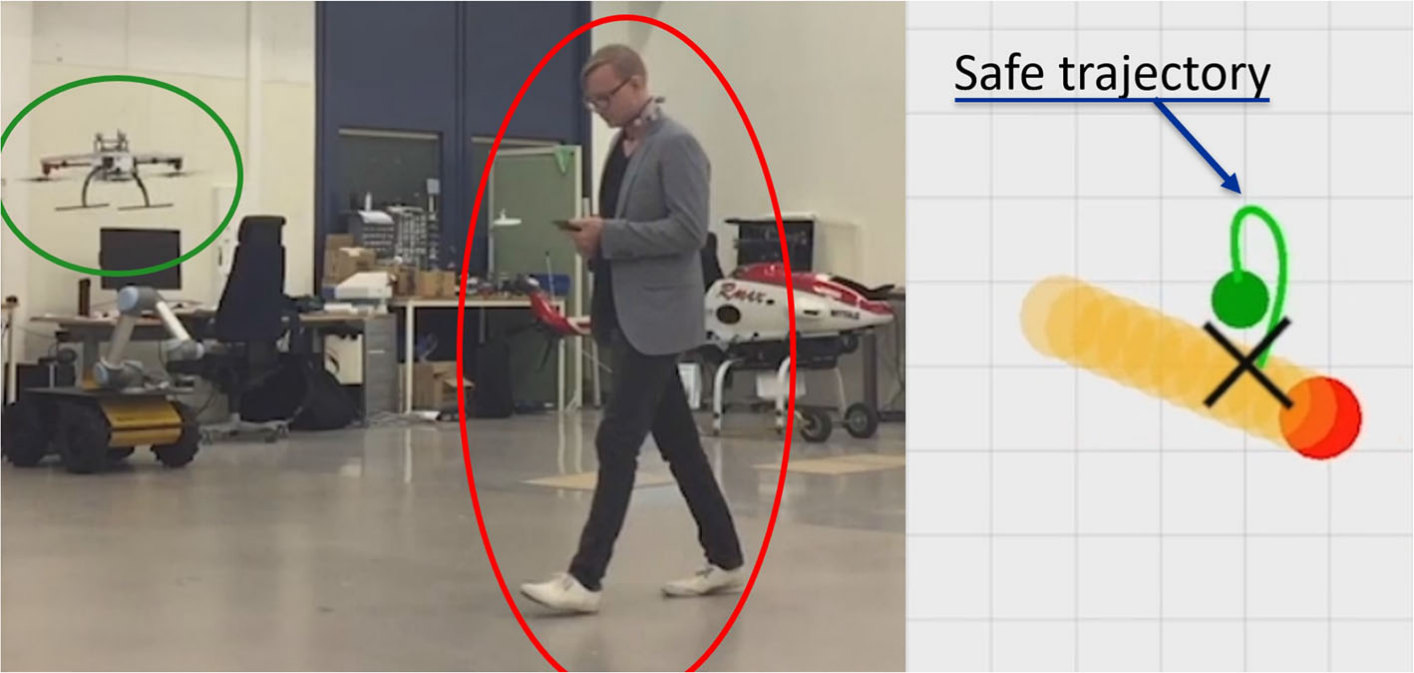
Testing a learned MPC controller that is safe under motion uncertainty

An example of MPC with a learned collision constraint that is safe (*p*=0.99) under motion uncertainty is depicted in Fig. 15. In this experiment, the collision constraint was learned in simulation and evaluated with a Vicon real-time positioning system. For the demo described in Section 5, the WARA-PS research infrastructure was later used and leveraged to extend this approach to safe online learning with only on-board sensing.

Several other research directions were also explored on the topic of safe planning in public environments populated by people. In [53] we considered learning computationally cheaper neural network approximations of safe policies *π*_NN_(***x***)≈*π*(***x***) for use on smaller embedded systems such as nano-quadcopters. By using a constraint-aware imitation learning approach [53], the quadcopters could maintain the required safety levels for the local avoidance problem, with performance increased to more than an order of magnitude faster. An advantage of neural network approximations is that they require a fixed amount of computation, where the performance vs. compute trade-off can be directly addressed just by changing the size of the network.

**Fig. 16:**
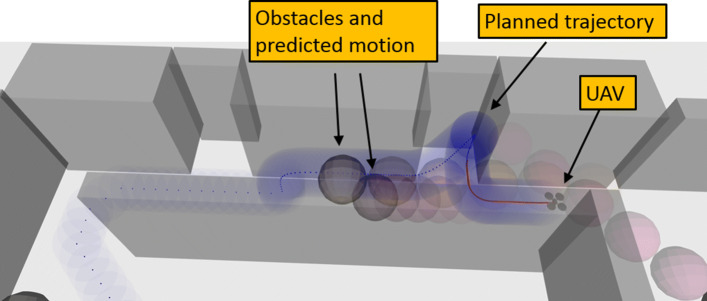
Motion planning in complex environments with people

In [54], local avoidance via MPC was extended to planning in more complex dynamic 3D environments such as that depicted in Fig. 16. The approach relies on a lattice approximation to a trajectory planning problem where the free space is time-dependent. Using a receding-horizon multi-resolution representation, the addition of a wait state allows for representation of temporal aspects while preserving the regularity of the lattice.

**Fig. 17:**
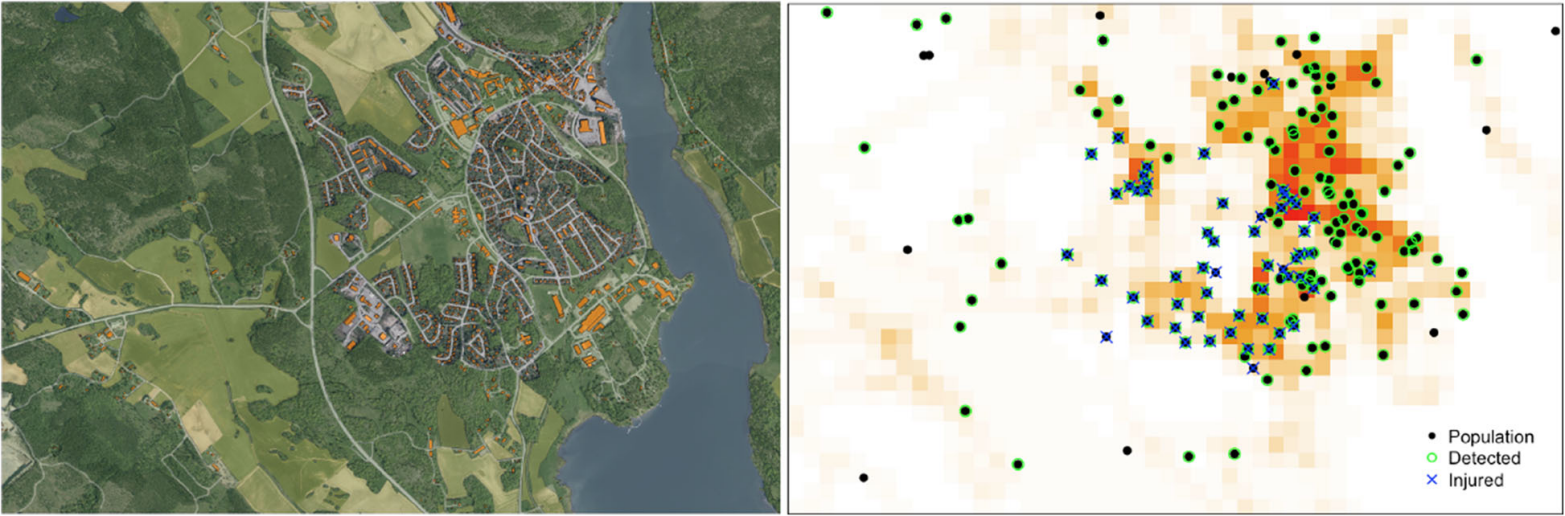
Informed aerial search of victims for disaster response missions. Left: Area map color-coded with GIS data. Right: Samples (thinned) from the structured spatial model

Finally, in [55] we consider real-time learning and search planning for automatic aerial victim search in disaster response missions. The proposed framework captures relevant problem desiderata in a probabilistic structured spatial model as shown in Fig. 17, which includes population density, probability of injury, as well as the probability of detection from the air. It allows informative priors from e.g. geographic information systems or cell-phone traffic data to be included, but it can also learn these individually via spatial point processes. Both probabilistic learning and search planning are computationally hard problems. For real-time learning we used an integrated nested-Laplace approximation tailored to such latent Gaussian fields. The search problem is a POMDP that directly minimizes victim harm. We use a deterministic belief-space approximation based on a receding-horizon Monte-Carlo tree-search with long-range macro actions and warm-starts. Instead of a separate task objective with constraints on safety, minimizing harm is the sole objective of this task.

## Collaborative autonomous ships/marine vessels (USV) experimentation

WARA-PS has access to a collection of unmanned marine vessels which are used individually for specific research problems but also participate in WARA-PS collaborative rescue scenarios. These platforms are part of the WARA-PS infrastructure accessible to all WASP participants.

### USV platforms

#### SAM and LoLo: SMaRC underwater platforms

**Fig. 18:**
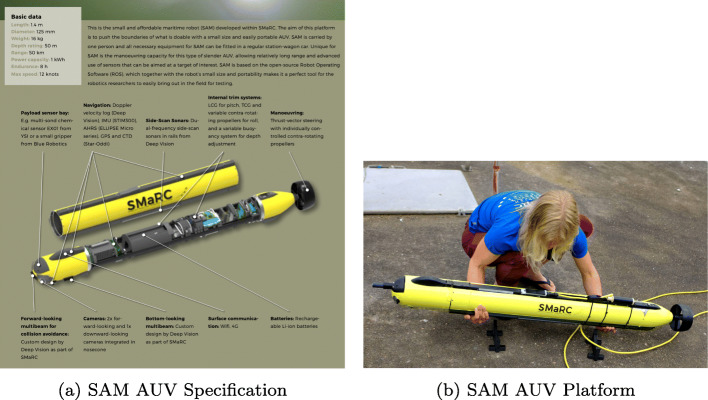
SAM underwater maritime robot developed by SMaRC

**Fig. 19:**
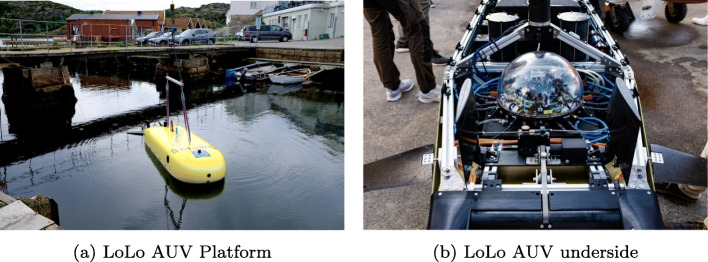
LoLo underwater maritime robot developed by SMaRC

The SMaRC Long-range and Long-endurance demonstrator (LoLo) and the Small and Affordable Maritime Underwater Robot (SAM) are both long-range and long-endurance maritime AUVs developed at SMaRC to promote hands-on research with underwater vehicles [56]. Figure 18 shows the SAM AUV platform and its specification. Figure 19 shows the LoLo AUV platform. LoLO has been used together with the Piraya USV in recent experimentation where LoLo can communicate and transfer data while underwater and in the vicinity of the Piraya. Both SAM and LoLo integrate nicely with the larger WARA-PS architecture due to their use of ROS (robot operating system) which is one of the middleware choices for the WARA-PS architecture.

#### Piraya platform

**Fig. 20:**
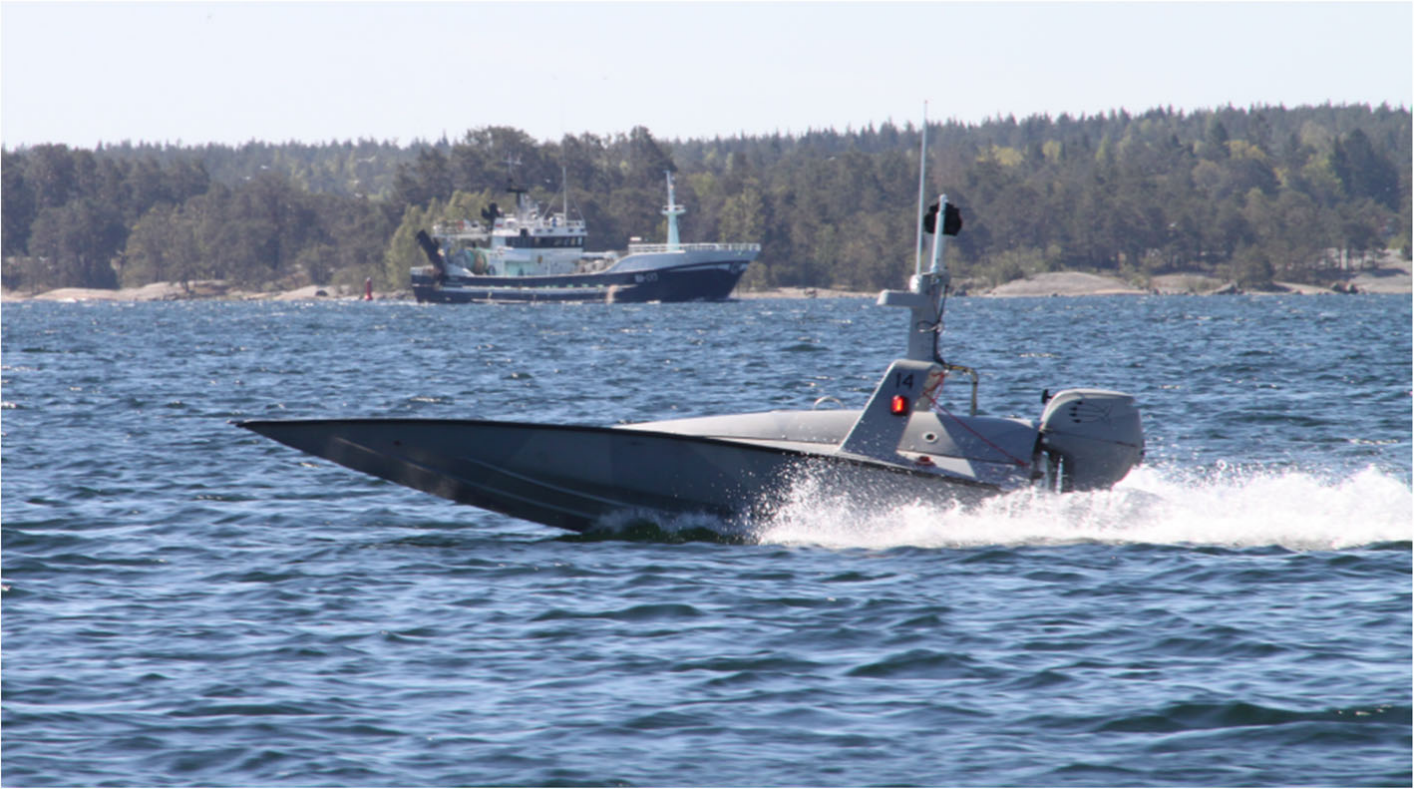
The Piraya by Saab Kockums

The Piraya (swedish for piranha) is an unmanned surface vehicle project under development by Saab Kockums in collaboration with the Swedish military. It is a small size boat with a 20 horsepower engine that runs autonomously. A novel feature of the Piraya is that several can be operated at the same time by a single person. Experimentation has been done using three Pirayas simultaneously operated by one operator. The Piraya has been integrated with the WARA-PS architecture and used for experimentation with collaborative robotics in sea rescue scenarios. Recent work has involved development of swarm algorithms for multi-platform navigation in challenging coastal scenarios requiring tight maneuvering. The Piraya can be equipped with a variety of sensors such as infrared cameras and hydrophones. One of the active research projects integrated cameras from Axis Communications AB, another industrial participant, for experimentation with image processing algorithms for navigation. Another project leashed DJI 100 platforms to Pirayas where they would autonomously follow the Piraya’s movements at sea. The Piraya is depicted in Fig. 20.

#### Combat boat 90 platform

**Fig. 21:**
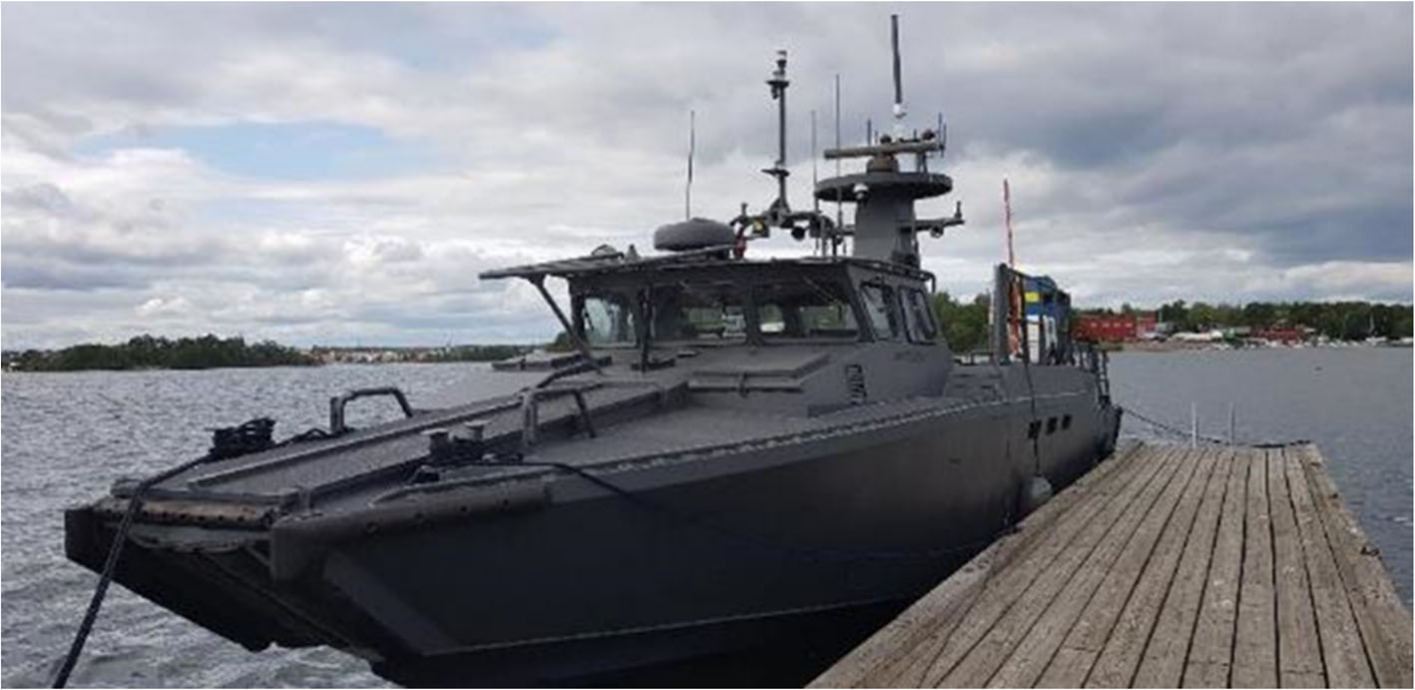
The CB90 by Saab Kockums

The Saab CB90 HSM is a combat boat commissioned for the Swedish Navy and sold world-wide. It has been developed by Kockums AB. It is 18 tons with a maximum displacement of 24.5 tons. The hull length is 16.3 meters. It has a cruise speed at sea of 42 knots using 2 x 900 HP, Scania Diesel V8 engines. The CB90, shown in Fig. 21, has been actively used in several research projects and demonstrations described in Sections 3.2.1, 4.2.1, and 5.1. Details for some of the sensors used in these research projects are provided in Table 1.

**Table 1:** Sensors used during field trials

Sensor	Description
Digital Compass*	Heading (Accuracy 0.5 ^∘^) - 1Hz
Speed Log*	STW (Accuracy 1% + 0.1knots) - 1Hz
Echosounder	Depth from surface to sea bed (Accuracy 0.1m) -
	1Hz
Magnetometer	Magnetic Intensity measured as a vector - 100Hz
360 ^∘^ camera	Provides visual image of the horizon around most
	of the ship. Can alternatively be multiple cameras.
	Images from 6 cameras were compiled into an
	image with a resolution of 16384 ×8192 - 15Hz

^∗^The digital compass and speed log could be exchanged with an INS

### Selected research with marine vessels

#### Robust GNSS-independent positioning through sensor fusion

For safe operation of a USV, it is vital to have a correct position. By knowing the position and compass direction, a sea chart can be used to determine a route to pass all static obstacles such as groundings and islands. The common approach for positioning is to use a Global Navigation Satellite System (GNSS), where the Global Positioning System (GPS) is the oldest and most used system. A loss of the GNSS signal, or if the ship is jammed or spoofed, can result in hazardous situations. A crew on a manned vessel can adapt to a situation like this, but an unmanned ship must have functionality beforehand for GNSS-independent positioning.

Terrain-Aided Navigation (TAN) is a widely used technique for GNSS-independent positioning. This technique often uses a particle filter, where each particle estimates the position of the vessel. First, thousands of particles are spread out around the initial position. Then in each iteration, all the particles are moved according to the ship’s velocity. The ship’s bottom depth is then measured and compared to each bottom depth reading in the map where particles are located. By comparing these bottom depths, weights, proportional to the likelihood of the position being correct, are created and assigned to each particle. In the last step of each iteration, the particles of the cloud are re-sampled, creating a new cloud that should correspond better with the true position. To get accurate and robust position estimations, an accurate map with enough resolution is preferred. Furthermore, the terrain must vary enough, so that the algorithm has a chance to discard particles with low weights.

There are several Autonomous Underwater Vehicles (AUV) using TAN, as AUVs can not use GNSS at all as they are submerged. Because there are not many areas in the world where high-resolution bathymetric maps have been created, an AUV mission is typically preceded by a bathymetric survey, where a ship measures the sea bottom and creates the needed high-resolution map. In order to avoid being limited by the low availability of high-resolution maps, our approach instead uses normal sea charts, which have much more sparse information, resulting in poorer position accuracy and robustness. To compensate for this, we use other information sources as well.

Earth is surrounded by a magnetic field, and in many areas, low-resolution maps are available to describe how this field varies. These measurements can be used by the particle filter in the same way as the bottom depth measurements. The particle filter described in [57] combined data from a high-accuracy INS with bottom depth information and magnetic intensity information. All data were simulated, and this resulted in a mean position error of 10.2m.

**Fig. 22:**
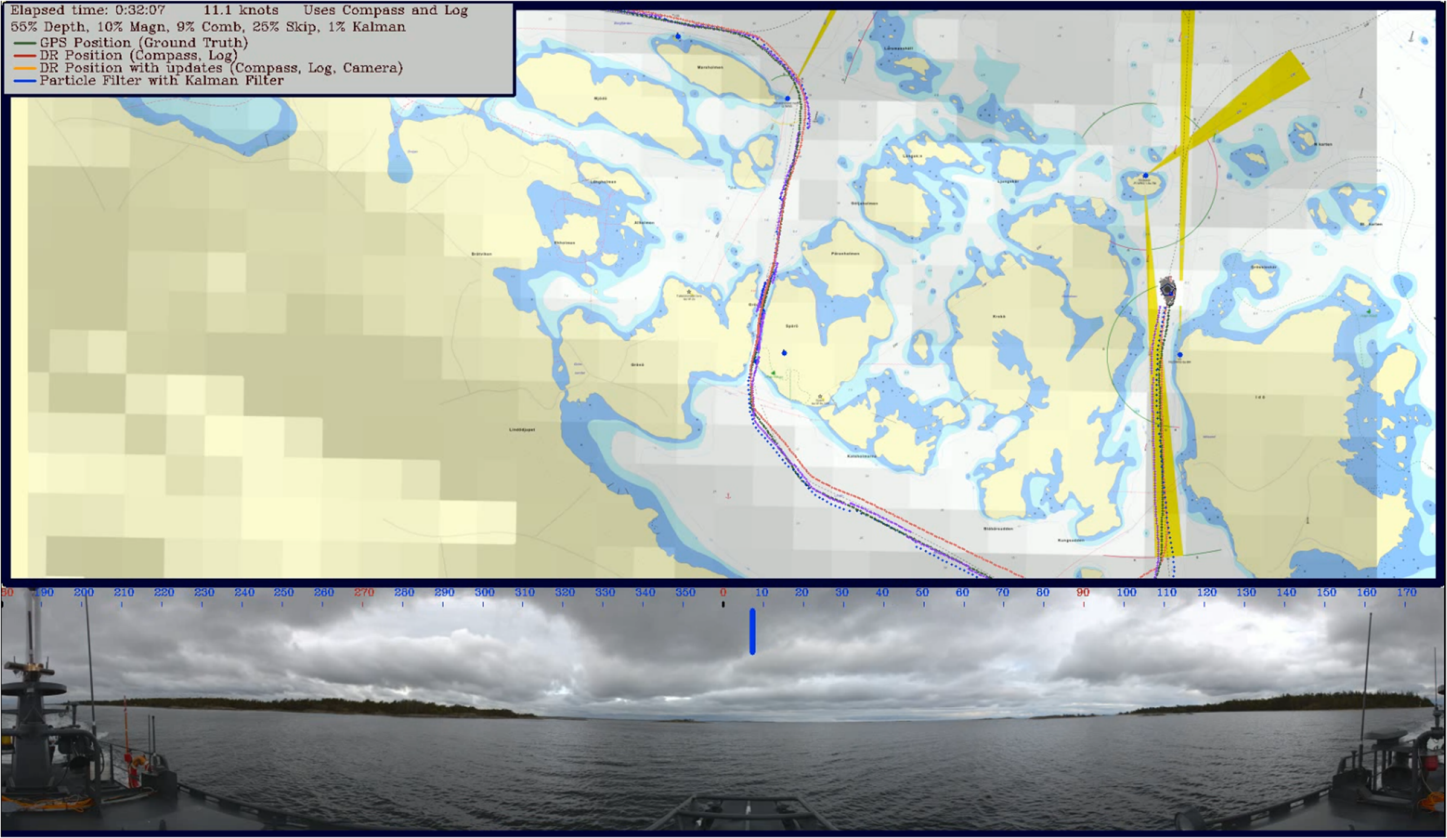
A screenshot of the GUI for the GNSS-independent positioning. The lower part shows a 360^∘^ image from a video recording the surrounding of the CB90. The upper part shows the sea chart, the current route, and the current position estimation by the particle cloud. The magnetic intensity anomaly map is overlaid on the image, seen as squares in various grey scale

The CB90 in WARA-PS was then used in [58] to evaluate the performance using real-world data. The GPS was used as ground truth, and instead of using an INS, the ship’s digital compass and speed log were used. Bottom depth and magnetic intensity were used, but also visual bearing measurements to surrounding landmarks. The GUI can be seen in Fig. 22. Simulations based on the real-world data showed that the proposed fusion mechanism provides accurate and robust positioning, and that the accuracy and robustness increases when using multiple data sources instead of depth or magnetic intensity individually.

#### USV VR teleoperation

While striving for full autonomy, we still have to assume that there might be occasions in which a human operator needs to assess a situation to provide a decision or even step in to remotely control a USV. We investigated how a graphical user interface (GUI) can be implemented to remotely supervise a small USV, while the communication throughput between the USV and the operator is limited. This is a realistic scenario for small affordable vessels.

Of particular interest was to see how the user’s situational awareness and cognitive load are affected when using such GUIs in comparison to using traditional ones. To answer these questions, we proposed a 3D-visualisation of the ship’s surroundings either on a computer screen or in a virtual reality (VR) setup [59, 60]. The perception of a 3D GUI in VR resembles how a human normally perceives the world, which is assumed to be beneficial for human-machine communication. The GUI design was based on ideas from the available research regarding manned ships to increase situational awareness while maintaining a low cognitive load, e.g., [61]. From these ideas, the assumption was that we could create a suitable GUI that would provide good situational awareness, and by that increase safety by

- Creating the GUI in 3D, and preferably presenting it in VR.
- Providing different views of the surrounding environment, optimized for various situations.
- Augmenting objects and information directly in the 3D world.
- Providing a 360 ^∘^ image of the real-world environment, so that the operator can compare the 3D world with the real world, to increase situational awareness and to manually detect objects.

**Fig. 23:**
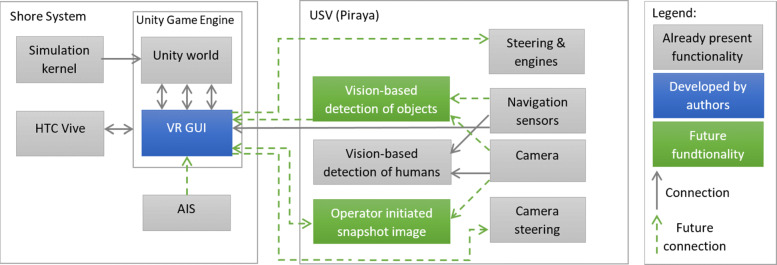
The VR GUI (or 3D GUI) is the main application where the interface is created for the operator. It presents the GUI in the Unity world, which is a 3D virtual environment (VE) with a virtual world positioned in the own ship’s location with surrounding ships simulated by the simulation kernel

We initially implemented three different GUIs and evaluated them in a small user study with 16 participants; one *Baseline GUI*, representing traditional navigation tools, one 3D tool presented on a laptop, called *3D GUI*, and one 3D tool presented in virtual reality (VR), called *VR GUI*. The implementation was done in Unity 3D [62], which is a development tool normally used for making 2D and 3D games. A 3D world (called *Unity World*), developed by Saab Kockums [5], was used as a foundation for the GUI, for which an overview is given in Fig. 23. A USV, also produced by Saab Kockums, has been used for initial testing.

We found that in particular the VR GUI improved the test subjects’ situational awareness and ability to detect and handle potentially harmful situations significantly in comparison to using a setup that resembled traditional tools (our *Baseline GUI*). Overall, we could confirm our hypothesis that an interface based on a 3D visualisation in VR would be a suitable tool to provide a remote operator with the necessary overview regarding a USV’s surroundings [59].

**Fig. 24:**
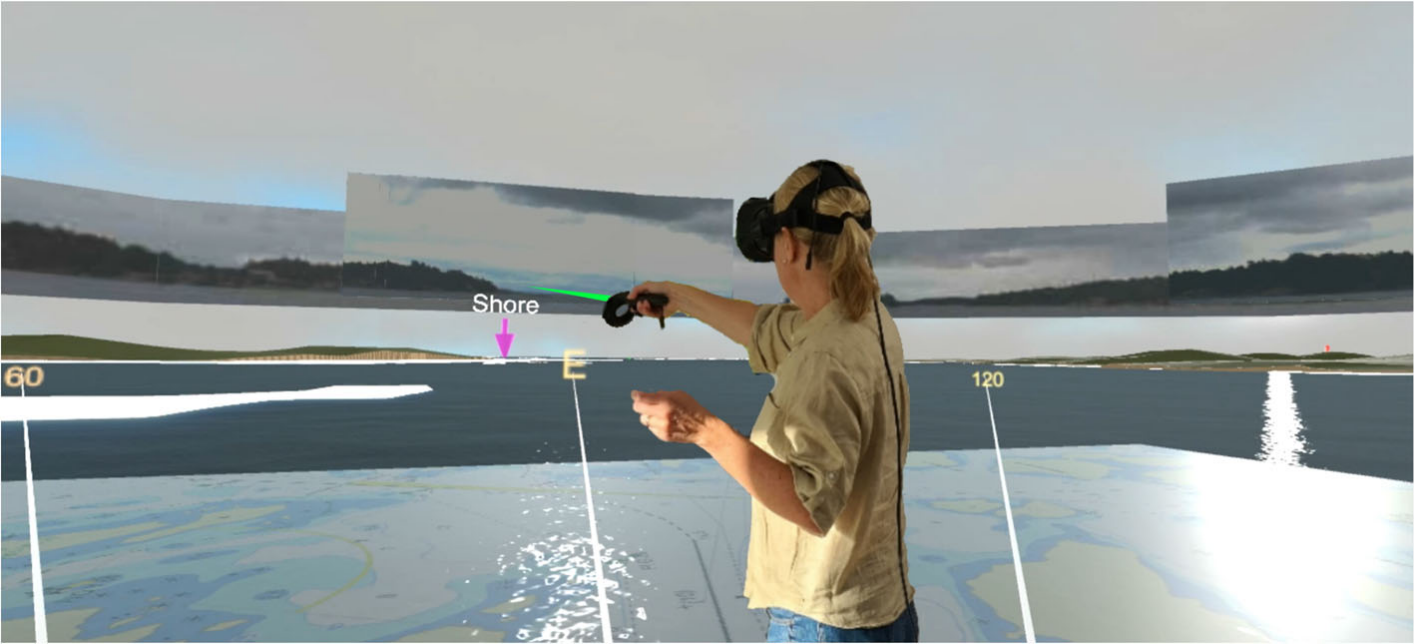
The user points towards the same object in the 360 ^∘^ image and the virtual world. This information can then be used to support the positioning algorithm, presented in Section 4.2.1

Based on these findings, we enhanced the initial GUI implementation to incorporate functions for supporting the positioning application described in Section 4.2.1 with bearings to landmarks. We also extended it so it could use real-world data from a field-trial in the WARA-PS site in Västervik, where navigation data and video were recorded from a 360 ^∘^ camera. An actual real-world user evaluation was not possible to conduct due to the Covid-19 pandemic. We provided the 360 ^∘^ images with relatively low quality (to limit bandwidth usage) to our test subjects to see whether they could detect (given or own) landmarks to support the positioning application and how this affected their experience with the system. An example of the GUI, together with one of the participants from the user-study, is seen in Fig. 24. We found that this extension to the GUI was very suitable to both provide another information (sensory) channel to the positioning system, and to enhance the users’ experience regarding the overall understanding (awareness) of the situation the vessel was in at any given time [60].

#### Semi-automated image annotation in marine environments

As indicated in Fig. 23 we assume visually observable landmarks to be a suitable source of information to align a map with reality. This is obviously true for human operators even in a VR-based setup, as we could confirm with our evaluation described in the previous section. However, also for (semi-)autonomous systems, visual information can be considered for GPS-independent navigation and positioning purposes. While there are quite many (annotated) data sets and annotation tools suitable for, among others, machine learning approaches in the area of autonomous vehicles on land ([63, 64], for example), there are relatively few starting points to work with image classification approaches in maritime environments. To mitigate that, we investigated the possibility to create a semi-automated tool for image annotation based on image sets gathered in the previously described trial and data collection runs in WARA-PS [65]. With the help of relatively general object detection and tracking approaches in combination with specific information for sea marks given in regular sea charts according to maritime standards from the International Hydrographic Organization (IHO) [66] and International Association of Marine Aids to Navigation and Lighthouse Authorities (IALA) [67], we created and evaluated a pipeline that can process relatively large data sets within considerably shorter time than this would take based on manual labour to produce an annotated data set for further use in learning based approaches to image processing in maritime environments.

**Fig. 25:**
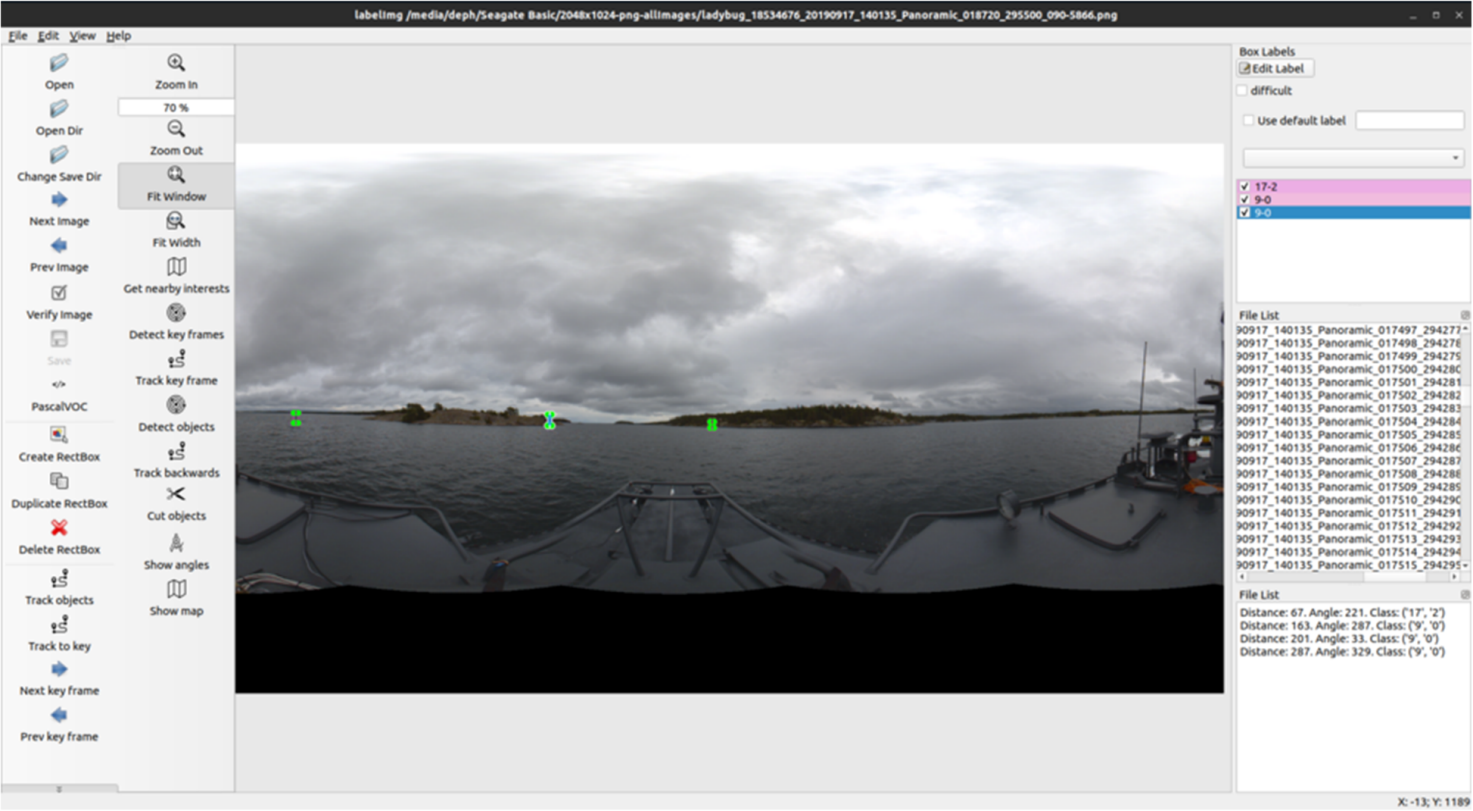
Final version of the annotation tool

Our processing pipeline included the following steps:

- Data alignment (images and position information from the data collection runs came in different frequencies and needed to be aligned)
- Transformation of all coordinate data to ECEF (Earth-centred, earth-fixed) format that is commonly used in GPS [68]
- Perform object detection and identify images (key frames) in which relevant objects occur at all. Keep those images for further processing. We evaluated several common approaches like YOLOv4 [69] and variants of R-CNN [70, for example] to receive suggestions for relevant objects in bounding boxes
- Track the objects over a number of frames starting with the identified key frames. We evaluated several tracking approaches such as Boosting [71], MIL [72], KCF [73], TLD [74], MedianFlow [75], Mosse [76], and CSRT (OpenCV implementation of CSR-DCF [77])
- Align information from detection and tracking steps with that given in the sea chart and propose an annotation for the found and tracked object
- Visualise the proposal (and original image sequence) in a graphical user interface (GUI) based on the open source annotation tool LabelImg [78], through which a human user can confirm hypotheses and disambiguate problematic cases. The GUI allows also to adjust the mode of annotation and provides general control over the annotation process. Figure 25 shows the final version of the GUI.

As indicated above we evaluated several combinations of approaches to object detection and tracking, and found that YOLOv4 for object detection / recognition and CSRT or KCF for tracking performed best overall, given the circumstances of a data set that was relatively unbalanced regarding the types of sea marks observed during the data collection. With this setup we performed further evaluation of the actual annotation process, to see how much we could speed up the annotation of a large amount of images without compromising regarding the quality compared to manual annotation.

Despite a somewhat imperfect performance of the detection and tracking components, we can state that our approach allows to speed up the process of image annotation in maritime environments significantly, as summarised in Table 2. The categories *easy* and *difficult* refer to differences in visibility of a specific object caused by distance and motion of the boat, for example. With *varied* we indicate that consecutive images are not necessarily stemming from the same sequence, but might come from various parts of the data set.

**Table 2:** Time to annotate a set of images manually and semi-automatically

Type	Images	Time (h)	Images/min	Avg time (s)
**Manual**	300	1:05:03	4.61	13.01
Easy	100	0:12:05	8.28	7.25
Difficult	100	0:14:07	7.12	8.47
Multiple objects	100	0:38:51	2.57	23.31
**Manual (Varied)**	154	1:11:10	2.164	27.73
**Semi-Automatic**	19890	3:07:15	106.22	0.56
Annotated	8646	2:59:20	48.21	1.24
Easy	2363	0:42:24	55.73	1.08
Difficult	3062	1:38:00	31.24	1.92
Multiple objects	1367	0:25:49	52.95	1.13

Our results showed that there is clearly room for improvement mainly regarding the tools for detection and tracking of relevant objects. However, despite relatively poor overall performance of these components in our pipeline, we still found that our approach supports image annotation quite well, mainly since all needed information is presented through the same GUI, allowing to quickly adjust erroneous proposals made by the automatic annotation tool. Thus, we believe to contribute the area of image processing for GPS-independent navigation and positioning at sea, by providing the means for training classifiers to recognise specific types of sea marks (or even specific instances).

## Selected WARA-PS research demonstrations

Each year, WARA-PS has an annual workshop that brings together researchers, students, companies and media to highlight progress in research and applications. During the workshops, time is dedicated for various field robotic experimentation and demonstrations in the Gränsö operational environment. Here the intent is to push state-of-the-art in among other topic areas, collaborative robotics and human/robot interaction. Both companies and universities show successes and challenges in this area where integration of many of the WARA-PS platforms in combination with human operators is demonstrated in the context of emergency rescue scenarios. This section highlights a number of successful demonstrations previously shown in addition to their research context.

### Autonomous drone landing experimentation

**Fig. 26:**
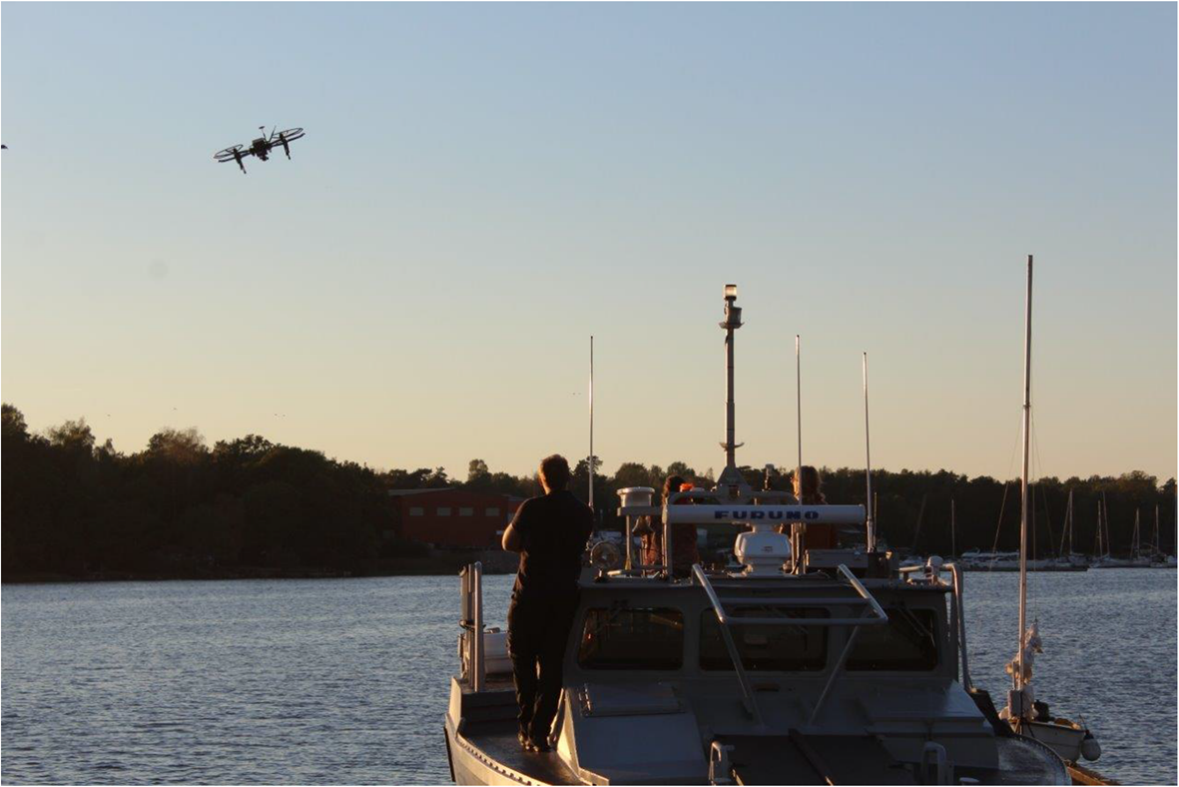
The quadcopter and CB90 boat during a landing trial

The autonomous landing of the quadcopter on top of a moving USV has been considered in several different experiments and tests within WARA-PS. Testing of the landing platform and the relative positioning system have been performed using the modified CB90 craft, see Fig. 26. For testing the cooperative MPC algorithms, including the real-time VH-MPC algorithm, tests have been performed using the real quadcopter in outdoors tests, landing on a virtual boat that can be simulated to move according to some reference trajectory.

**Fig. 27:**
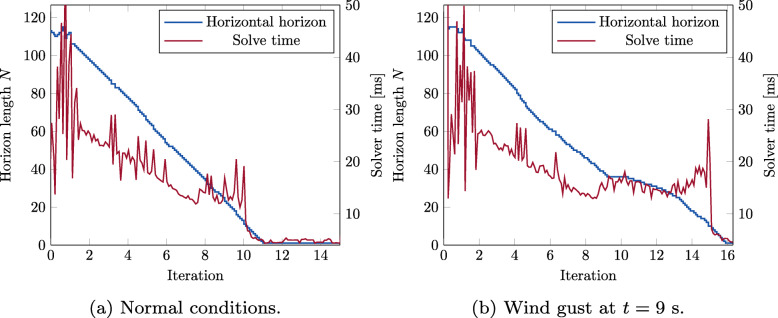
The horizon of the horizontal VH-MPC (left axis) in the outdoors experiments, with the horizontal solver time (right axis). The upper image shows the result when no significant wind disturbances are present, and the lower figure shows the case when a large wind gust disturbs the drone around *t*=9 s. The horizon is adapted to the new conditions and starts decreasing again when a landing is possible. The illustrated experiments were performed with a sampling time *dt*=0.1 s

By testing the algorithms in real environments subject to nonlinear dynamics, disturbances, communication delays, and other effects, we got a comprehensive evaluation of the control performance. All autonomous landing tests were performed in mild to moderate wind conditions. Figure 27 illustrates the effectiveness of the VH-MPC algorithm. The VH-MPC algorithm is implemented in real-time using the OSQP solver [45]. The solver has been modified to make it possible to update the factorization of the KKT conditions without having to redo the entire factorization. In Fig. 27(a), we see the VH-MPC algorithm applied to the quadcopter landing under nominal conditions. Since the sampling time is 0.1 s, the initial predition of the maneuver time is 10-12 s. It can be seen from the figure that this corresponds well to the final maneuver time, which is around 11 s. In Fig. 27(b), a landing from the same initial conditions is repeated but this time there was a wind gust disturbing the landing at *t*=9 s. The controller handles this unexpected disturbance very well, and the horizon is adapted to the changing circumstances.

### Self-learning of human-drone safety constraints

This demo builds on research in learning safety constraints for navigation that was presented in Section 3.2.2. The WARA infrastructure was leveraged to demonstrate this using a DJI Matrice 100 quadcopter that was assigned ground-level way points to fly to while having to avoid any people moving in the same area. The idea was to extend prior work in two ways, i) *online* learning of safety constraints, and ii) incorporation an on-board perception stack in-the-loop instead of using Vicon’s external positioning functionality.

**Fig. 28:**
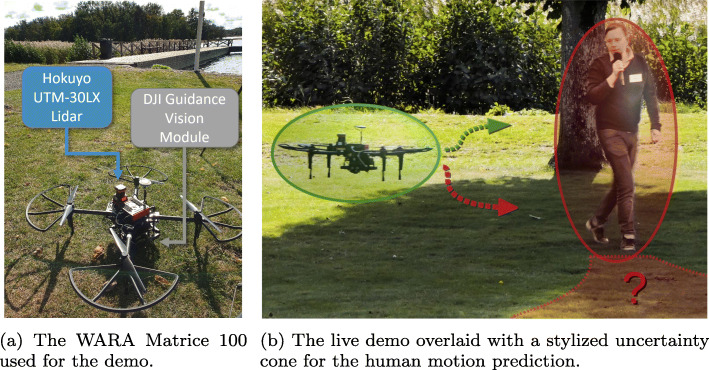
Live obstacle avoidance demonstration in the Gränsjö operational environment during a WARA-PS Workshop

The robustness requirements are quite high on the perception stack in order to enable a quadcopter to robustly detect and navigate among moving people. Since this demo required full autonomy, low rates of both false negatives and false positives are required, which pure camera-based approaches still struggle with. A Hokuyo lidar was used instead and it was mounted horizontally for reliable distance measurements around the quadcopter, as seen in Fig. 28(a). As GPS may not be reliable close to obstacles such as trees, buildings or possibly even people, a DJI Guidance vision sensor was also employed for optical flow and stereo-based ground plane extraction.

Since reliable segmentation of static and moving parts in complex 3D scenes is still an open problem, the demo was based on the local learning-based avoidance of [48]. This approach only relies on a sparse obstacle representation, where obstacles are encoded as geometric constraints on an MPC program. The obstacle detection was achieved via Euclidian clustering and ground plane extraction. Any motion was then detected and tracked using constant velocity Kalman filters on the cluster centers.

A determinized NMPC controller was implemented as a compact mathematical program in CasADi [79], where auto-differentiation and the built-in SQP solver delivered adequate performance. The moving obstacles were represented by ellipsoid safety constraints centered on their mean prediction $\hat {\boldsymbol {x}}_{o,t}$ for each obstacle *o* and time *t* over the MPC planning horizon. Since obstacle constraints make the resulting feasible region in Eq. 7 non-convex, random restarts were employed so as to ensure that the controller could escape any local minima as in earlier work.

Suitable safety constraints were then learned online using constrained Bayesian optimization with the real world in the loop. The objective used was the time to reach its waypoints, and the safety constraint used was on maintaining a minimum distance to moving obstacles. Both are uncertain functions of the safety parameters and modelled as Gaussian processes. Safe online learning was enforced by requiring that the safety constraint be fulfilled during the optimization process. The quadcopter starts with a parameter prior or set of conservative parameters that are known to be safe. Then, during the Bayesian optimization it selects parameters to test, not only based on their expected improvement of the objective, but also such that the probability of violating the safety constraint, the minimum distance to people, is held below *p* as in Eq. (6).

A depiction of the demo with a stylized representa- tion of motion uncertainty for a moving person is shown in Fig. 28(b). This is often difficult to model in practice. The actual safety distance needed for the quadcopter additionally depends on the agility of the platform, as well as uncertainty and latency in its control and perception layers. As most of these are also difficult to model, attempting to directly learn safety constraints from real-world experiments can be more accurate. While the safety constraints can be arbitrary functions of problem state, due to time constraints for the demo, only one parameter was used – a simple isometric size of safety ellipsoids, held constant over the prediction horizon.

**Fig. 29:**
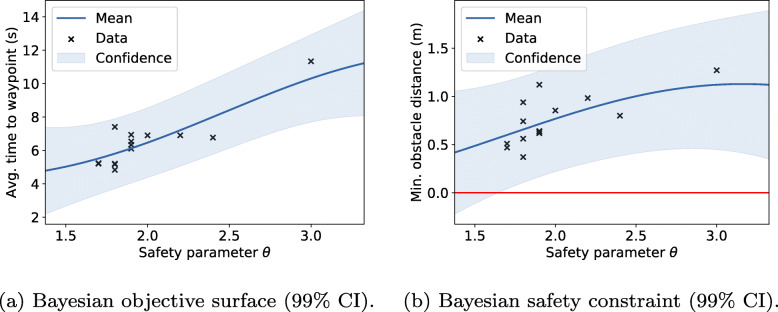
Example of safe online Bayesian learning for one safety parameter *θ*

An example of online safety parameter optimization is shown in Fig. 29, where it attempts to minimize its traversal time while satisfying the safety constraint Pr(dist(***x***_robot_,***x***_obst_)>0)>0.99, where the minimum distance to obstacles is greater than zero with *p*=0.99.

The demo provided some valuable insights and generated several ideas for future research directions, including i) the online learning approach was successful as a proof of concept, but more could be done to better scale it to larger problem instances, ii) while the focus of our work here was on the decision and control side, much research also remains on the perception side for robust navigation in more complex dynamic environments, and finally iii) while most work in learning and motion planning is done in simulation, knowing the limitations of the perception side of the problem can also lead to insights in how to design better simulators or solutions tailored to a specific application.

## Conclusion

As has been described in this paper, WARA-PS is a major effort and a large investment in a state-of-art platform for computing, communication and control of unmanned vehicles. The objectives are to collect data, to perform realistic outdoor experiments and to demonstrate a collaborative autonomous search and rescue system. This work is done in close collaboration between academia and companies. We have described some selected research projects on autonomous systems, but the scope of WARA-PS is much broader including AI for decision making, machine learning, software systems, cloud and edge technology, computer vision, visualization and human machine interaction. We strongly believe that realistic large scale experiments are necessary to do major scientific breakthroughs in the area of intelligent systems, in addition to expediently transferring knowledge from academia to industry.

WARA-PS continually addresses new and more challenging research topics and complex real-life field-robotic scenarios, for example, rescue operations under adverse conditions, such as high winds, snow and severe weather. Robustness, reliability and resilience are of course most important in safety critical search and rescue operations where unforeseen contingencies abound. These topic areas, crucial to the success of robotics in actual rescue scenarios and robotics at large, offer challenges where much additional research and innovative engineering is required. The nature of the WARA-PS arena allows for valuable contributions to these issues and these topics will be pursued.

The impact and application of current research, experiments and demonstrations are much larger than the current demos, and can be applied to a multitude of public safety applications. Autonomous drone systems for surveillance and urban transport are currently very active areas both in industry and in academia. For example, there is a tremendous amount of interest in the development of personal air transportation to avoid the congestion in current urban 2D transport infrastructures.

The first batch of WASP/WARA-PS PhD projects has just finished, but there are currently over 300 active PhD students within the WASP program. Many of them are industrial PhD students performing their research projects within companies and in cooperation with participating Swedish universities. WASP has launched new WARA-X arenas in, for example, software technology, industrial robotics and media and natural language processing. The ambition is that these arenas will create beneficial synergies, not only within each arena, but between arenas. This is particularly important from both research and pragmatic perspectives since, the issues targeted are highly complex and multi-disciplinary and require extensive collaboration across disciplines.

## Data Availability

Some data sets and materials are accessible at the WASP-WARA Portal and may be provided upon request. Not applicable. No custom code has been submitted.

## Footnotes

1 A more detailed description of the delegation process is described in [24]. This description provides a brief summary based on that reference.
